# Radiomic and Genomic Machine Learning Method Performance for Prostate Cancer Diagnosis: Systematic Literature Review

**DOI:** 10.2196/22394

**Published:** 2021-04-01

**Authors:** Rossana Castaldo, Carlo Cavaliere, Andrea Soricelli, Marco Salvatore, Leandro Pecchia, Monica Franzese

**Affiliations:** 1 IRCCS SDN Naples Italy; 2 University of Warwick Coventry United Kingdom

**Keywords:** prostate cancer, machine learning, systematic review, meta-analysis, diagnosis, imaging, radiomics, genomics, clinical, biomarkers

## Abstract

**Background:**

Machine learning algorithms have been drawing attention at the joining of pathology and radiology in prostate cancer research. However, due to their algorithmic learning complexity and the variability of their architecture, there is an ongoing need to analyze their performance.

**Objective:**

This study assesses the source of heterogeneity and the performance of machine learning applied to radiomic, genomic, and clinical biomarkers for the diagnosis of prostate cancer. One research focus of this study was on clearly identifying problems and issues related to the implementation of machine learning in clinical studies.

**Methods:**

Following the PRISMA (Preferred Reporting Items for Systematic Reviews and Meta-Analyses) protocol, 816 titles were identified from the PubMed, Scopus, and OvidSP databases. Studies that used machine learning to detect prostate cancer and provided performance measures were included in our analysis. The quality of the eligible studies was assessed using the QUADAS-2 (quality assessment of diagnostic accuracy studies–version 2) tool. The hierarchical multivariate model was applied to the pooled data in a meta-analysis. To investigate the heterogeneity among studies, *I*^2^ statistics were performed along with visual evaluation of coupled forest plots. Due to the internal heterogeneity among machine learning algorithms, subgroup analysis was carried out to investigate the diagnostic capability of machine learning systems in clinical practice.

**Results:**

In the final analysis, 37 studies were included, of which 29 entered the meta-analysis pooling. The analysis of machine learning methods to detect prostate cancer reveals the limited usage of the methods and the lack of standards that hinder the implementation of machine learning in clinical applications.

**Conclusions:**

The performance of machine learning for diagnosis of prostate cancer was considered satisfactory for several studies investigating the multiparametric magnetic resonance imaging and urine biomarkers; however, given the limitations indicated in our study, further studies are warranted to extend the potential use of machine learning to clinical settings. Recommendations on the use of machine learning techniques were also provided to help researchers to design robust studies to facilitate evidence generation from the use of radiomic and genomic biomarkers.

## Introduction

Prostate cancer (PCa) is the second most diagnosed cancer worldwide in men [1,2]. To guarantee cancer-specific survival, early detection of PCa is essential at a treatable stage. The most common method to diagnose PCa is via transrectal ultrasonography (TRUS) [3]. The rapid development of medical imaging techniques and modalities has demonstrated great value in the screening, diagnosis, treatment response measurement, and prognosis evaluation of PCa. In particular, radiomic investigation, defined as computationally extracting quantitative image features for the characterization of disease patterns [4], has been intensively applied to tumor detection, localization, staging, aggressiveness assessment, treatment decision-making assistance, and patient follow-up in PCa [5] .

More recently, multiparametric magnetic resonance imaging (mpMRI) has been demonstrated to be a better radiomic biomarker than systematic TRUS biopsy, achieving high diagnostic accuracy and becoming a clinical routine investigation for suspected PCa patients [6,7]. The second version of the Prostate Imaging Reporting and Data System (PI-RADS-V2) was updated in regard to minimum technical acquisition parameters and image interpretation [8]. It describes a standard prostate mpMRI protocol that combines anatomical T2-weighted images with functional sequences, that is, diffusion-weighted imaging (DWI) or dynamic contrast-enhanced (DCE) sequences.

Alongside radiomic investigation, there are numerous Food and Drug Administration–approved genomic biomarkers underlying the biomolecular functions most strongly associated with clinical outcomes. In fact, a major focus of personalized medicine has been the biomolecular characterization of tumors by integrating genomics into clinical oncology to identify unique druggable targets and generate higher-order tumor classification methods that can support clinical treatment decisions [9]. They are mainly used to decide whether biopsy screening is necessary and whether patients require primary treatment (such as radical prostatectomy or radiation therapy) [10]. The combination of biopsy screening and evaluation of the Gleason score still remains the most widely accepted grading system in the evaluation of prostatic adenocarcinoma [11]. The Gleason grading system is based on a morphologic continuum of architectural dedifferentiation and is directly correlated with response to therapy and mortality rate. However, novel biomarker tests that can potentially detect PCa from blood, urine, tissue, and semen samples continue to be investigated. Prostate-specific antigen (PSA) is the most commonly used biomarker for the management of PCa [12]. Increased PSA density has been shown to be associated with increased risk of PCa compared to healthy or benign prostatic hyperplasia patients [13]. The Prostate Health Index and 4Kscore utilize isoforms of PSA and its precursors to help risk-stratify patients with an abnormal PSA level. In addition, microRNAs have an important role during tumor progression, and their combination with PSA serum can improve prediction of PCa status [14-16]. Other proposed biomarkers that belong to various classes of biological compounds, including proteins and metabolites, have shown to be noninvasive methods with high diagnostic potential [17].

Over the last decade, the landscape for PCa detection tools has expanded to include novel biomarkers, clinical information, genomic assays, and noninvasive imaging tests. The prospect of detecting PCa using readily available clinical and demographic health information is a potentially innovative part of improving screening practices [18].

In this scenario, machine learning (ML) is helping researchers in identifying and discovering new biomarkers to detect PCa. ML is a branch of artificial intelligence (AI) techniques based on the development and training of algorithms by learning from data and the performance of predictions. ML methods are able to improve and learn over time in a more efficient way than classical statistical approaches [19]. Therefore, ML has been widely used in radiology and recently in the field of bioinformatics [6,20]. A recent field of ML, deep learning (DL), is based on artificial neural networks, which offer superior problem-solving capabilities applied to large heterogenous data sets [20,21]. Specifically, ML allows the integration or combination of different layers of data, such as those from medical images, laboratory results, clinical outcomes, biomarkers, and other biological features, for better prognostication and stratification of patients toward personalized medicine [22,23]. However, the accuracy of such algorithms can be highly impacted by the complex workflows adopted to develop and generalize such ML algorithms [24,25]. High heterogeneity is expected, as ML problems are usually regarded as black boxes, and the consideration of all possible risk factors and transformation is tremendously difficult [26,27]. Moreover, there are no clear guidelines on how to develop ML approaches for medical studies.

Therefore, this study aimed to suggest an integrated estimate of the accuracy for use of ML algorithms in detecting PCa through a systematic review and meta-analysis of the available studies. Due to the internal heterogeneity of ML algorithms, subgroup analyses helped in investigating the diagnostic capability of ML systems and highlighting the sources of bias and common pitfalls to avoid in order to assure reproducibility among studies. Subgroup analyses were mainly based on the model choice, model development, and validation methods to identify potential covariates that could influence the diagnostic performance of ML.

This review helps to support ML studies in rising up the pyramid of evidence. In fact, we identify and discuss recurrent factors that hinder the uptake of these studies in clinical settings.

To the best of the authors’ knowledge, there are no systematic review and meta-analysis studies evaluating the performance and estimating the current status of existing approaches on PCa detection. Therefore, this study aims to fill the gap in the existing literature and gather recommendations on ML model development to achieve robust results to automatically detect PCa.

## Methods

We conducted and reported this meta-analysis in accordance with PRISMA (Preferred Reporting Items for Systematic Reviews and Meta-Analyses) guidelines [28]. Two researchers (RC and MF), who were blinded to the articles’ author information, conducted the study inclusion, data extraction, and assessment of the risk of bias independently. A third author (CC) was consulted in case of disagreements.

### Search Strategy

The PubMed, Scopus, and OvidSP (ie, Embase) databases were searched to identify studies evaluating the accuracy of radiomic, clinical, and genomic biomarkers in the diagnosis of PCa. The following criteria were used to limit the research: papers published in the last 5 years (from 2015 to 2020) to guarantee homogeneity among radiomic studies, as the new protocol (PI-RADS) for mpMRI was updated in 2015 [8]; study on adult humans (ie, not animals); language (English); and full-text publications. The search took place on February 24, 2020. The reference lists of the included studies were checked, and the authors were contacted if required. The search strategy and queries for each search database are presented in Table S1 in Multimedia Appendix 1.

An author (RC) retrieved the initial search results and removed duplicates via Excel (Microsoft). Subsequently, another author (MF) manually searched for and removed any remaining duplicates. Finally, RC and MF independently screened the studies by title, abstract, and keywords, after which the full texts of the selected studies were assessed by inclusion and exclusion criteria. The main considerations for study inclusion were if machine learning was fully applied in distinguishing individuals or lesions with clinically diagnosed PCa from controls and if the study assessed the accuracy of such applications. Detailed inclusion and exclusion criteria are reported in Table S2 in Multimedia Appendix 1.

### Data Extraction and Outcomes of Interest

After the evaluation was completed, two authors extracted the following information from the selected literature: literature data—the first author, publication date, study population, number of patients, study design, and data collection; basic research information—age, Gleason score, and PSA level, where possible; information regarding the reference standard used in individual studies; definitions of positive and negative PCa (PCa positive and control) and methodologies to distinguish individuals or lesions with PCa from the control group; specific methodologies to process and classify data for use in machine learning algorithms; and the sensitivity, specificity, and, if available, true-positive (TP), true-negative (TN), false-positive (FP), and false-negative (FN) rates.

The authors independently graded the quality of the eligible studies using the quality assessment of diagnostic accuracy studies–version 2 (QUADAS-2) tool [29]. The full process is provided in the supplementary materials in Multimedia Appendix 1.

### Meta-analysis Paper Inclusion Criteria and Subgroup Analysis

For radiomic analysis, due to the very low number of included studies investigating central gland and transition zone (TZ) prostate tumors, only studies investigating the peripheral zone were included in the meta-analysis. This was also due to the fact that central gland and TZ prostate tumors have significantly different quantitative imaging signatures [30], and they could have highly biased the final results.

Due to the low number of studies employing 3D volumes of interest (VOIs) to extract quantitative features, only studies delineating 2D regions of interest (ROIs) were included in the meta-analysis to reduce the risk of bias. This was mainly due to the fact that significant differences were found between prediction performance when using 3D VOIs and that when using 2D ROIs [31]. If studies investigated several diagnostic imaging techniques via ML, only classification models using mpMRI sequences were included in the meta-analysis.

To reduce heterogeneity among the selected studies, subgroup analyses were carried out for radiomic and genomic studies due to their intrinsic differences in data acquisition, analysis, and feature extraction. Radiomic subgroup analyses helped to investigate the role of the mpMRI biomarker in detecting PCa via ML, whereas genomic subgroup analyses were carried out to understand the role of genomic biomarkers in detecting PCa via ML.

Several covariates suitable for subgroup analysis were identified during the review process where the individual peculiarities of the studies, which may affect the outcome, were investigated.

The included studies were investigated if they explored a patient- or lesion-based model, validation approaches (cross-validation, hold-out approach or external validation, or no validation), ML algorithms (regression-based model, tree-based model, or deep learning algorithms), whether the studies used a DL or ML approach, or whether the employed data set was balanced or unbalanced. For genomic studies, the use of different specimens (ie, urine, serum, semen, and tissue) was also investigated in a subgroup analysis. One study [17] investigated both urine and serum specimens separately; therefore, ML performance was included for both predictors in the meta-analysis.

In case a study investigated multiple ML algorithms, only the method achieving the highest area under the curve (AUC) was included in the meta-analysis, as AUC is a good estimator of ML performance.

### Statistical Analysis and Software Tools

This meta-analysis was conducted via the Open Meta-Analyst Software tool, and statistical significance was expressed with 95% CIs. Pooled estimates for sensitivity and specificity with the corresponding 95% CIs were used to determine the accuracy of machine learning for detecting PCa in radiomic and genomic studies. From these data, we generated a hierarchical summary receiver operating characteristic curve (HSROC) and coupled forest plots by random-effects model. Heterogeneity among studies was assessed by calculation of the inconsistency index (*I*^2^) and evaluation of the Cochran *χ*^2^ test (*Q* test). An *I*^2^ of ≥50% and *P*<.001 indicated substantial between-study heterogeneity. The TP/FP/TN/FN values were extracted or calculated from each independent study. A correction factor of 0.5 was added if any of the TP/FP/TN/FN rates reported a value of 0, in order to prevent zero cell count problem [32].

In our meta-analysis, a multivariate random-effects model was used to consider both within- and between-subject variability and threshold effects [33]. The HSROC curve was specified by pooled sensitivity and specificity point. Attempts were made to resolve the heterogeneity by performing a subgroup analysis [34].

## Results

### Literature Search

**Figure 1:**
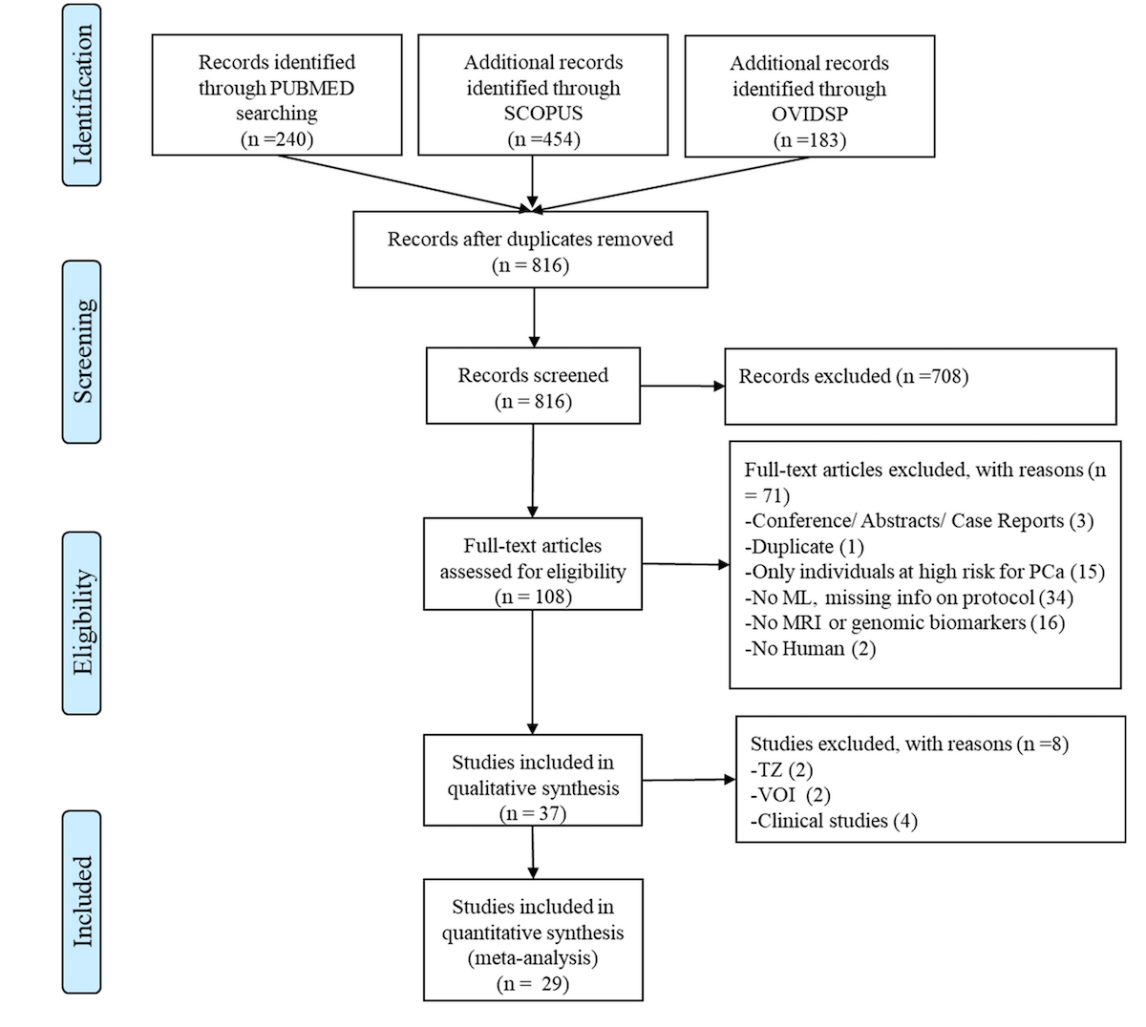
PRISMA (Preferred Reporting Items for Systematic Reviews and Meta-Analyses) flowchart of literature search: included/excluded titles, abstracts, and full papers. ML: machine learning; MRI: magnetic resonance imaging; PCa: prostate cancer; TZ: transition zone; VOI: volume of interest.

According to the search strategy described above, 877 titles were identified in PubMed, Scopus, and OvidSP. After removing duplicates, 816 titles were considered. Of these, 708 were excluded after reading of the abstracts because they did not meet the inclusion criteria. From the remaining 108 full-text articles, 71 were removed due to the exclusion criteria. Finally, 37 full texts were included in the qualitative analysis, and 29 studies were considered appropriate for inclusion in the meta-analysis. A flowchart of the literature search is shown in Figure 1.

The distribution of the risk of bias evaluated via the QUADAS-2 tool for the included studies is presented in the supplementary materials (Figure S1 in Multimedia Appendix 1).

### Characteristics of the Included Studies

The publication years ranged from 2015 to 2020 to guarantee homogeneity among radiomic studies, as the new PI-RADS was updated in 2015 [8]. All patients were diagnosed with PCa by biopsy. The main characteristics of the studies are reported in Table 1. The extracted raw data are presented in Tables S3 and S4 in Multimedia Appendix 1.

**Table 1:** Characteristics of 37 studies included in the systematic review.

Characteristics			Studies, n	Patients (average over the number of studies), n
**Study type**				
	Prospective		8	2210 (276.25)
	Retrospective		29	6414 (221.17)
**Data set type**				
	Private data set		33	7760 (235.15)
	Public database (SPIE-AAPM-NCI^a^ PROSTATEx challenge)		2	399 (199.5)
	Mixed (private and public) data set		2	465 (232.5)
**Classification algorithms**				
	Random forest		4	1621(405.25)
	Regression-based models		20	4678 (233.9)
	Partial least squares discriminant analysis (PLS-DA)		2	180 (90)
	Linear discriminant analysis (LDA)		1	53
	Support vector machine (SVM)		2	65 (32.5)
	Classification and regression tree (CART)		1	67
	Artificial neural networks (ANNs)		2	1012 (506)
	Deep neural networks (DNNs)		1	195
	Convolutional neural networks (CNNs)		3	696 (232)
	Deep learning: SNCSAE^b^		1	57
**Predictor type**				
	Multiparametric MRI^c^		20	5058 (252.9)
	**Genetic or molecular biomarker**		13	3132 (240.92)
		Urine	6	930 (155)
		Serum	3	901 (300.3)
		Semen	2	108 (54)
		Tissue	2	800 (400)
	Clinical data		4	2812 (703)
**Validation method**				
	Internal validation		29	6540 (225.52)
	External validation		3	1380 (460)
	Internal and external validation		1	364
	Unknown		5	704 (140.8)

^a^SPIE-AAPM-NCI: International Society for Optics and Photonics–American Association of Physicists in Medicine–National Cancer Institute.

^b^SNCSAE: stacked nonnegativity constraint sparse autoencoders.

^c^MRI: magnetic resonance imaging.

### Quantitative Analysis (Meta-analysis)

Of the final 37 papers, 29 were considered for the meta-analysis. Eight studies were excluded to reduce heterogeneity among the studies. Of those, 2 studies were excluded because they extracted radiomic features from VOIs [35,36], and 2 studies [37,38] were excluded because they only focused on detecting TZ tumors. Due to the low number of studies investigating TZ tumors, a comparative assessment of the results for the peripheral zone, central gland, and TZ was not possible.

Studies [18,39-41] employing only clinical information were excluded because a minimum sample of 5 studies is recommended for a meta-analysis [34,42]. In fact, 5 or more studies are needed to reasonably achieve power from random-effects meta-analyses [43].

#### Radiomic

All the included studies for the radiomic analysis are reported in Table 2. A total of 4438 independent samples were inspected from 16 studies with sensitivity and specificity ranging from 0.62 to 0.99 and 0.51 to 0.98, respectively.

Multivariate meta-analysis via the HSROC model was assessed for all the studies (Figure S2 in Multimedia Appendix 1). The pooled sensitivity and specificity were 0.815 (95% CI 0.410-0.999) and 0.828 (95% CI 0.424-0.999), respectively.

The calculated heterogeneity values for pooled sensitivity and specificity were 84% and 79% (*P*<.001), respectively; therefore, a random-effects model was adopted to generate coupled forest plots (Figure S3 in Multimedia Appendix 1).

##### Subgroup Analysis

**Figure 2:**
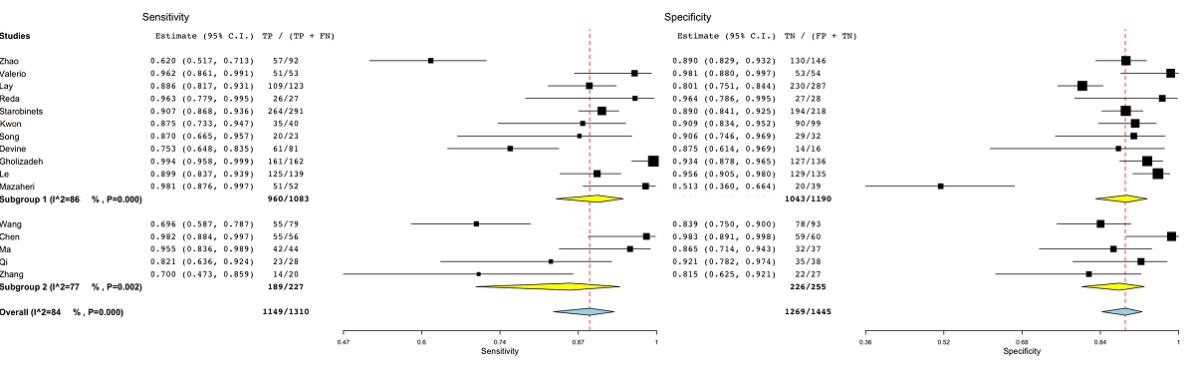
Subgroup analysis for the model-based covariate in radiomic studies. Subgroup 1: lesion-based models; subgroup 2: patient-based models. FN: false-negative; FP: false-positive; TN: true-negative; TP: true-positive.

To resolve the heterogeneity, subgroup analysis was conducted for different covariates. The subgroup analysis per model-based covariate is shown in Figure 2. Subgroup 1 included the studies that employed a lesion-based ML approach. Those studies [44-54] employed multiple lesions for each patient enrolled in the study. Subgroup 2 gathered those studies [55-59] that enrolled two distinct groups (PCa and controls) and employed a patient-based ML approach. The heterogeneity in the subgroups was greater than 70% (subgroup 1: *P*<.001, subgroup 2: *P*=.002*)*.

**Table 2:** Accuracy measures of radiomic studies for the systematic review.

Study, year	Model basis^a^	Patients, n	Total sample (PCa+, PCa-)^b^	Crossval^c^/ split/none	ML^d^ methods^e^	TP,^f^ n	FN,^g^ n	FP,^h^ n	TN,^i^ n	Sen^j^ (lower-upper)	Spe^k^ (lower-upper)
Zhao, 2015 [44]	LB	71	238 (92, 146)	120 (60, 60)	ANN	57	35	16	130	0.620 (0.517-0.713)	0.890 (0.829-0.932)
Valerio, 2016 [45]	LB	53	106 (53, 53)	None	LDA	51	2	1	53	0.962 (0.861-0.991)	0.981 (0.880-0.997)
Lay, 2017 [46]	LB	224	410 (123, 287)	Crossval	RF	109	14	57	230	0.886 (0.817-0.931)	0.801 (0.751-0.844)
Reda, 2017 [47]	LB	18	53 (26, 27)	Crossval	SNCSAE	26	1	1	27	0.963 (0.779-0.995)	0.964 (0.786-0.995)
Starobinets, 2017 [48]	LB	169	509 (291, 218)	Crossval	LR	264	27	24	194	0.907 (0.868-0.936)	0.890 (0.841-0.925)
Wang, 2017 [36]	PB	172	172 (79, 93)	Crossval	DCNN	55	24	15	78	0.696 (0.587-0.787)	0.839 (0.750-0.900)
Le, 2017 [52]	LB	364	913 (463, 450)	275 (139, 135)	multimodal CNN	125	14	6	129	0.899 (0.837-0.939)	0.956 (0.905-0.980)
Kwon, 2018 [49]	LB	204	191 (36, 155)	Crossval	LASSO LR	35	5	9	90	0.875 (0.733-0.947)	0.909 (0.834-0.952)
Song, 2018 [50]	LB	195	547 (261, 286)	55 (23, 32)	DNN	20	3	3	29	0.870 (0.665-0.957)	0.906 (0.746-0.969)
Chen, 2019 [56]	PB	381	381 (182, 199)	155 (55, 60)	LR	55	1	1	59	0.982 (0.884-0.997)	0.983 (0.891-0.998)
Devine, 2019 [51]	LB	65	97 (81, 16)	Crossval	LR	61	20	2	14	0.753 (0.648-0.835)	0.875 (0.614-0.969)
Gholizadeh, 2019 [54]	LB	11	297 (161, 136)	Crossval	SVM	161	1	9	127	0.994 (0.958-0.999)	0.934 (0.878-0.965)
Ma, 2019 [58]	PB	81	81 (44, 37)	None	LR	42	2	5	32	0.955 (0.836-0.989)	0.865 (0.714-0.943)
Mazaheri, 2019 [53]	LB	67	170 (102, 68)	91 (52, 39)	CART	51	1	19	20	0.981 (0.876-0.997)	0.513 (0.360-0.664)
Qi, 2019 [57]	PB	199	199 (85, 114)	66 (28, 38)	LR	23	5	3	35	0.821 (0.636-0.924)	0.921 (0.782-0.974)
Zhang, 2019 [59]	PB	140	140 (60, 80)	Crossval	RF	14	6	5	22	0.700 (0.473-0.859)	0.815 (0.625-0.921)

^a^LB: lesion-based model; PB: patient-based model.

^b^PCa: prostate cancer.

^c^Crossval: cross-validation techniques.

^d^ML: machine learning.

^e^ANN: artificial neural networks; LDA: linear discriminant analysis; RF: random forest; SNCSAE: stacked nonnegativity constraint sparse autoencoders; LR: logistic regression; DCNN: deep convolutional neural networks; LASSO: least absolute shrinkage and selection operator; DNN: deep neural networks; SVM: support vector machine; CART: classification and regression tree.

^f^TP: true-positive.

^g^FN: false-negative.

^h^FP: false-positive.

^i^TN: true-negative.

^j^Sen: sensitivity.

^k^Spe: specificity.

**Figure 3:**
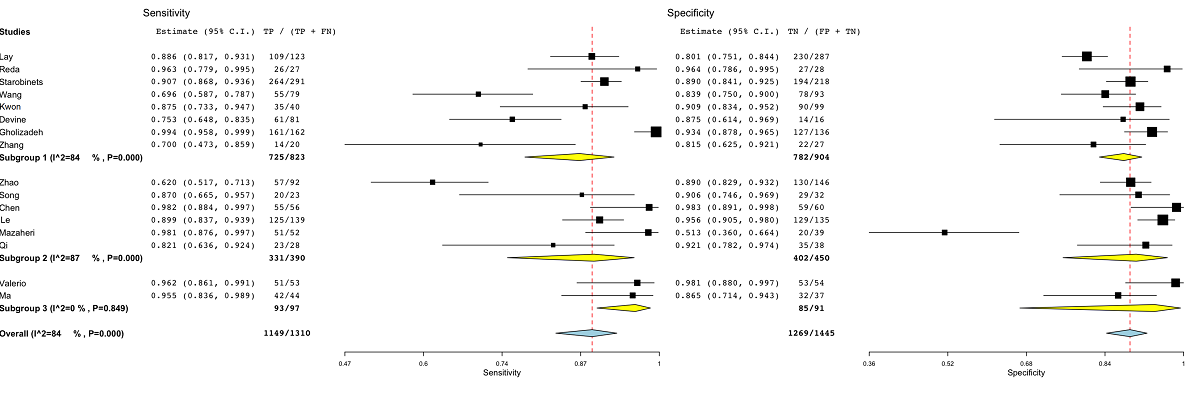
Subgroup analysis for the validation covariate in radiomic studies. Subgroup 1: internal cross-validation; subgroup 2: hold-out approach or external validation; subgroup 3: no validation. FN: false-negative; FP: false-positive; TN: true-negative; TP: true-positive.

Figure 3 shows the subgroup analysis among studies that employed internal cross-validation techniques (subgroup 1) [46-49,51,54,55,59], split validation approaches (subgroup 2) [44,50,52,53,56,57], and no validation (subgroup 3) [45,58]. The heterogeneity for subgroups 1 and 2 was around 80% (*P*<.001).

**Figure 4:**
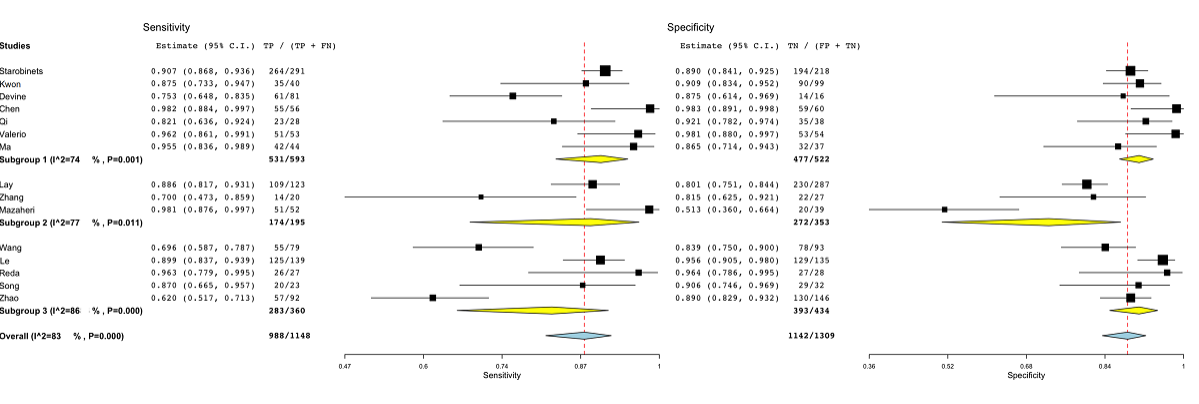
Subgroup analysis for the machine learning algorithm covariate in radiomic studies. Subgroup 1: regression-based models; subgroup 2: tree-based models; subgroup 3: deep learning methods. FN: false-negative; FP: false-positive; TN: true-negative; TP: true-positive.

Figure 4 shows the subgroup analysis for regression-based models (subgroup 1) [45,48,49,51,56-58], tree-based models (subgroup 2) [46,53,59], and DL methods (subgroup 3) [44,47,50,52,55]. One study was not included [54], as it was the only study employing a support vector machine model. The heterogeneity among groups oscillated between 74% and 86% (subgroup 1: *P*=.001, subgroup 2: *P*=.01, subgroup 3: *P*<.001).

**Figure 5:**
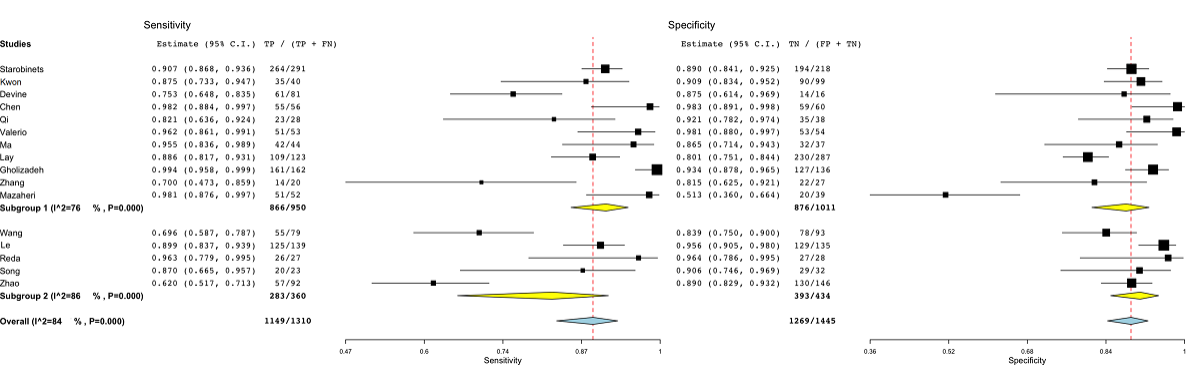
Subgroup analysis for the machine learning or deep learning covariate in radiomic studies. Subgroup 1: machine learning–based models; subgroup 2: deep learning methods. FN: false-negative; FP: false-positive; TN: true-negative; TP: true-positive.

The results of the subgroup analysis to discriminate among machine and deep learning methods are reported in Figure 5. Subgroup 1 included the studies [45,46,48,49,51,53,56-59] employing ML methods, whereas subgroup 2 comprised the studies [44,47,50,52,55] employing DL methods (based on artificial neural networks) such as convolutional neural networks and deep neural networks. The *I*^2^ statistics for subgroups 1 and 2 were 76% and 86% (*P*<.001), respectively.

**Figure 6:**
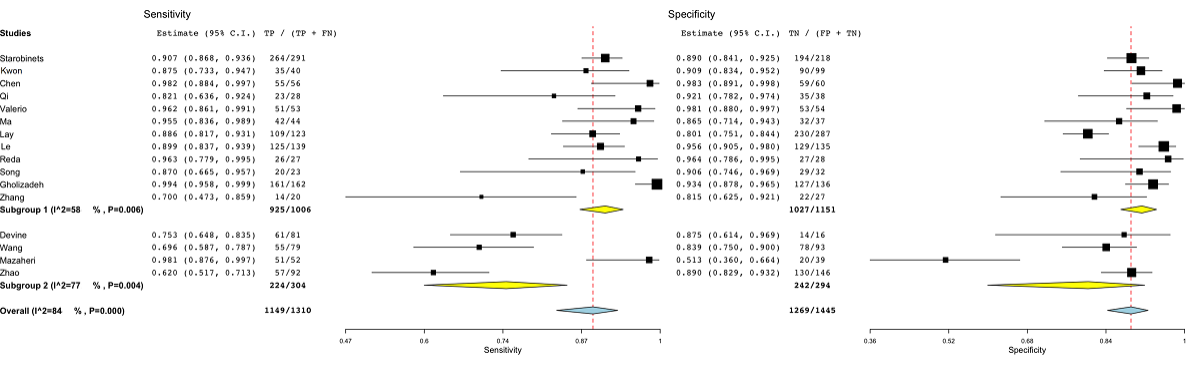
Subgroup analysis for the imbalance covariate in radiomic studies. Subgroup 1: balanced data sets; subgroup 2: unbalanced data sets. FN: false-negative; FP: false-positive; TN: true-negative; TP: true-positive.

Figure 6 shows the subgroup analysis based on whether the studies employed a balanced or unbalanced data set. A data set was defined as unbalanced if it had more than 30% of the total observations in one specific class rather than the other (PCa and controls) and did not apply any correction on performance (eg, synthetic minority oversampling technique [SMOTE] or voting techniques). The heterogeneity of subgroup 1 [36,44,51,53] was around 58% (*P*=.005). As a result, among the several covariates, the imbalance covariate was the only one by which the heterogeneity could be partially resolved.

**Figure 7:**
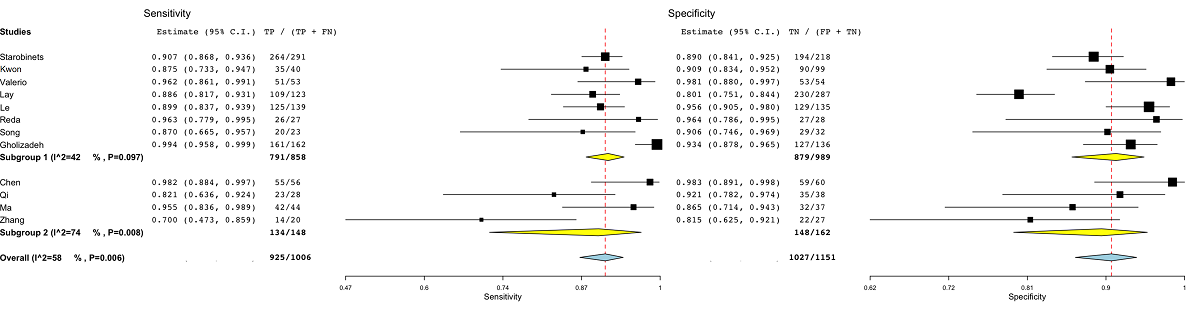
Subgroup analysis for the model-based covariate in a subset of radiomic studies. Subgroup 1: lesion-based models; subgroup 2: patient-based models. FN: false-negative; FP: false-positive; TN: true-negative; TP: true-positive.

Therefore, Devine et al [51], Wang et al [36], Mazaheri et al [53], and Zhao et al [44] were excluded from the coupled forest plot (Figure 7).

**Figure 8:**
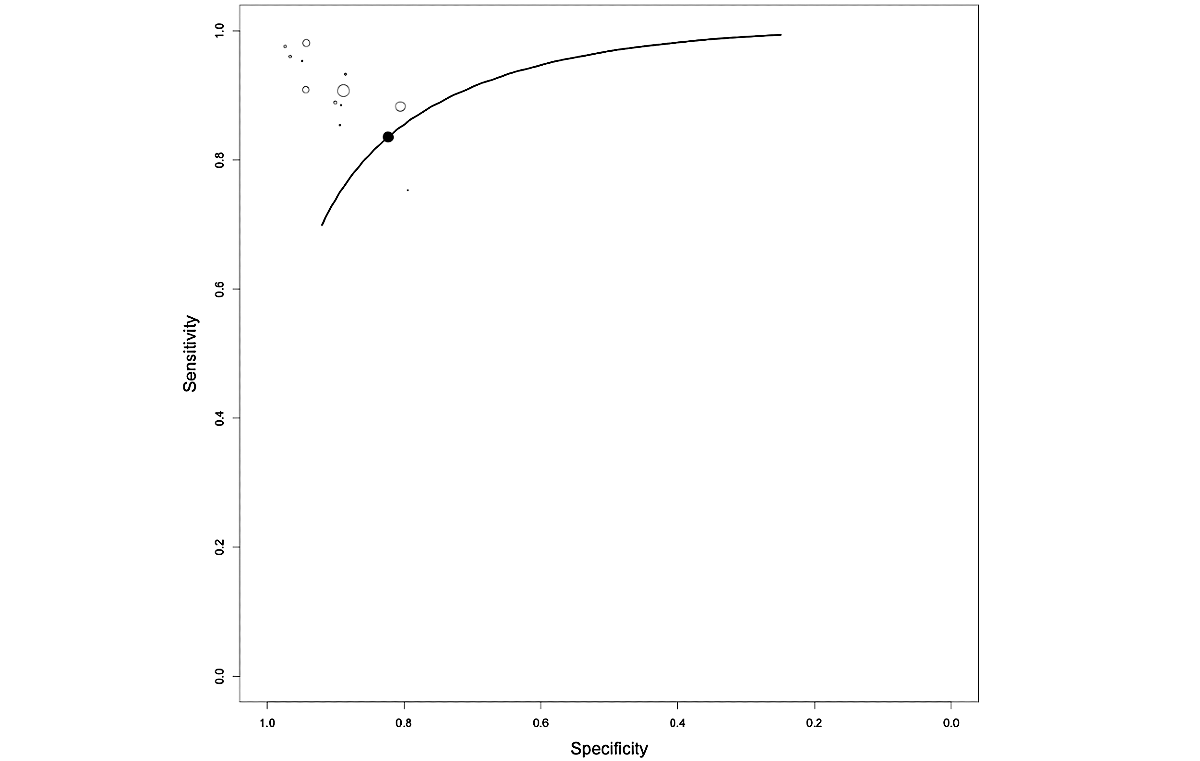
Overall hierarchical summary receiver operating characteristic curve (HSROC) for a subset of radiomic studies. HSROC was calculated for radiomic studies with low heterogeneity, excluding 4 studies [36,44,51,53].

Figure 8 shows the HSROC curve for the studies employing balanced data sets to automatically detect PCa. The pooled sensitivity and specificity were 0.808 (95% CI 0.38-0.999) and 0.831 (95% CI 0.41-0.999), respectively.

#### Genomic

All the included studies for the genomic analysis are reported in Table 3. A total of 3221 independent samples were inspected from 14 studies and included in the meta-analysis, with sensitivity and specificity ranging from 0.67 to 0.95 and 0.15 to 0.97, respectively.

An HSROC model was assessed for all genomic studies (Figure S4 in Multimedia Appendix 1). The pooled sensitivity and specificity were 0.883 (95% CI 0.541-0.999) and 0.734 (95% CI 0.330-0.999), respectively.

The calculated heterogeneity values for the pooled sensitivity and specificity were 73% and 92% (*P*<.001), respectively; therefore, a random-effects model was adopted to generate the coupled forest plots (Figure S5 in Multimedia Appendix 1).

##### Subgroup Analysis

**Figure 9:**
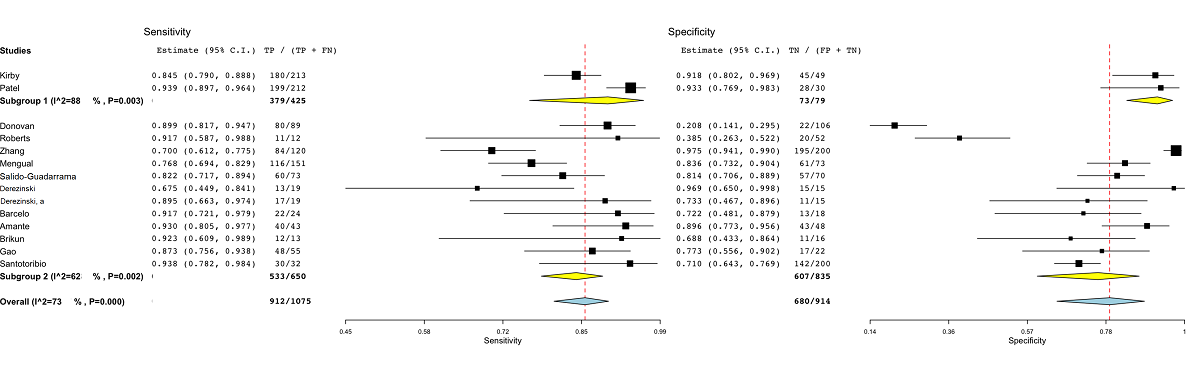
Subgroup analysis for the model-based covariate in genomic studies. Subgroup 1: lesion-based models; subgroup 2: patient-based models. FN: false-negative; FP: false-positive; TN: true-negative; TP: true-positive.

To resolve this heterogeneity, subgroup analyses were conducted for several covariates. The subgroup analysis for model-based covariates is shown in Figure 9. Subgroup 1 included the studies [60,61] that used malignant lesions and benign-adjacent tissue from PCa patients. Subgroup 2 gathered those studies [15-17,62-69] that enrolled two distinct groups (PCa and controls) and employed a patient-based ML approach. The heterogeneity for subgroup 1 was greater than 80%, whereas for subgroup 2 it was around 60%. However, subgroup 2 only included 2 studies.

**Table 3:** Accuracy measures of genomic studies for the systematic review.^a^

Study, year	Model basis^b^	Predictor	Patients, n	Total sample (PCa+, PCa-)^c^	Crossval^d^/ split/none	TP,^e^ n	FN,^f^ n	FP,^g^ n	TN,^h^ n	Sen^i^ (lower-upper)	Spe^j^ (lower-upper)
Donovan, 2015 [62]	PB	Urine	195	195 (89, 106)	None	80	9	84	22	0.899 (0.817-0.947)	0.208 (0.141-0.295)
Roberts, 2015 [16]	PB	Semen	66	66 (12, 54)	Crossval	11	1	32	20	0.917 (0.587-0.988)	0.385 (0.263-0.522)
Zhang, 2015 [63]	PB	Serum	580	580 (180, 400)	320 (120, 200)	84	36	5	195	0.7 (0.612-0.775)	0.975 (0.941-0.99)
Mengual, 2016 [64]	PB	Urine	224	224 (15, 73)	Crossval	116	35	12	61	0.768 (0.694-0.829)	0.836 (0.732-0.904)
Salido-Guadarrama, 2016 [15]	PB	Urine	143	143 (73, 70)	None	60	13	13	57	0.822 (0.717-0.894)	0.814 (0.706-0.889)
Dereziński, 2017 [17]	PB	Serum	89	89 (49, 40)	34 (19, 15)	13	6	0	15	0.675 (0.449-0.841)	0.969 (0.65-0.998)
Dereziński, 2017a [17]	PB	Urine	89	89 (49, 40)	34 (19,15)	17	2	4	11	0.895 (0.663-0.974)	0.733 (0.467-0.896)
Kirby, 2017 [60]	LB	Tissue	101	398 (286, 112)	262 (213, 49)	180	33	4	45	0.845 (0.79-0.888)	0.918 (0.802-0.969)
Barceló, 2018 [65]	PB	Semen	42	42 (34, 18)	None	22	2	5	13	0.917 (0.721-0.979)	0.722 (0.481-0.879)
Amante, 2019 [66]	PB	Urine	91	91 (43, 48)	Crossval	40	3	5	43	0.93 (0.805-0.977)	0.896 (0.773-0.956)
Brikun, 2019 [67]	PB	Urine	94	94 (42, 52)	29 (13, 16)	12	1	5	11	0.923 (0.609-0.989)	0.687 (0.433-0.864)
Gao, 2019 [69]	PB	Urine	183	183 (108, 75)	77 (55, 22)	48	7	5	17	0.873 (0.756-0.938)	0.773 (0.556-0.902)
Patel, 2019 [61]	LB	Tissue	699	795 (699, 96)	242 (212, 30)	199	13	2	28	0.939 (0.897-0.964)	0.933 (0.769-0.983)
Santotoribio, 2019 [68]	PB	Serum	232	232 (32, 200)	None	30	2	58	142	0.937 (0.782-0.984)	0.71 (0.643-0.769)

^a^All studies employed regression-based models.

^b^LB: lesion-based model; PB: patient-based model.

^c^PCa: prostate cancer.

^d^Crossval: cross-validation techniques.

^e^TP: true-positive.

^f^FN: false-negative.

^g^FP: false-positive.

^h^TN: true-negative.

^i^Sen: sensitivity.

^j^Spe: specificity.

**Figure 10:**
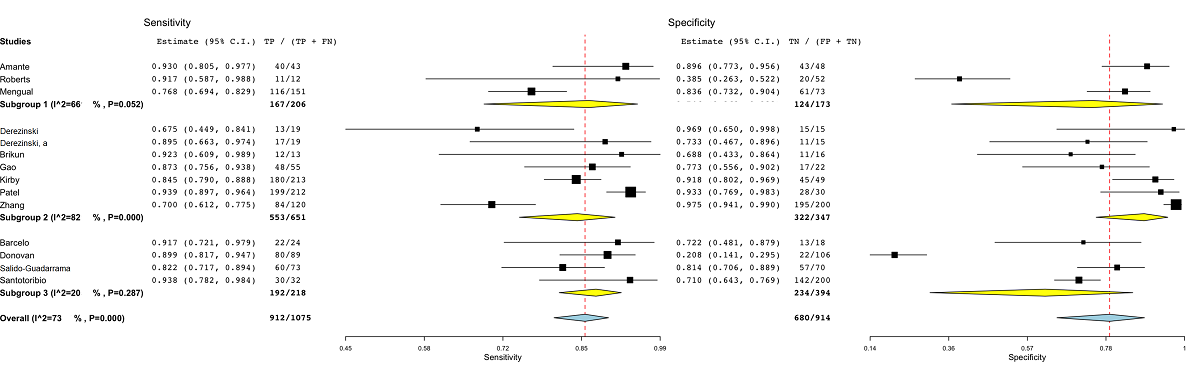
Subgroup analysis for the validation covariate in genomic studies. Subgroup 1: internal cross-validation; subgroup 2: hold-out approach or external validation; subgroup 3: no validation. FN: false-negative; FP: false-positive; TN: true-negative; TP: true-positive.

The subgroup analysis among studies that employed internal cross-validation techniques (subgroup 1) [16,64,66], split validation approaches (subgroup 2) [17,60,61,63,67,69], and no validation (subgroup 3) [15,62,65,68] is shown in Figure 10. For subgroups 1 and 2, the heterogeneity was greater than 50%. In subgroup 3, the heterogeneity was around 20%.

**Figure 11:**
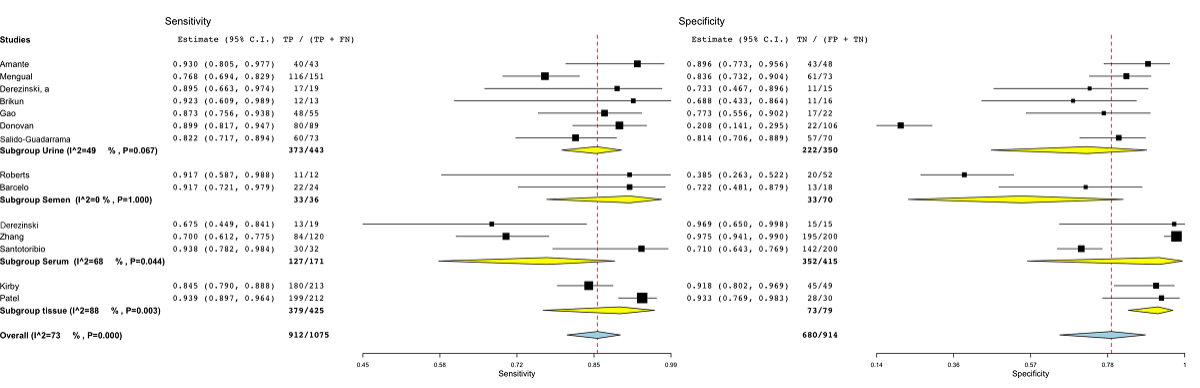
Subgroup analysis for the predictor covariate in genomic studies. FN: false-negative; FP: false-positive; TN: true-negative; TP: true-positive.

A subgroup analysis was also carried out based on the specimen used by the genomic studies (ie, urine [15,17,62,64,66,67,69], semen [16,65], serum [17,63,68], and tissue [60,61] biomarkers). The subgroup of studies investigating urine biomarkers to automatically detect PCa presented a lower heterogeneity than studies employing tissue and serum biomarkers and included more than 5 studies (Figure 11).

An inspection of ML algorithms among genomic studies was not possible because all the included studies employed a regression-based model (Table S4 in Multimedia Appendix 1).

**Figure 12:**
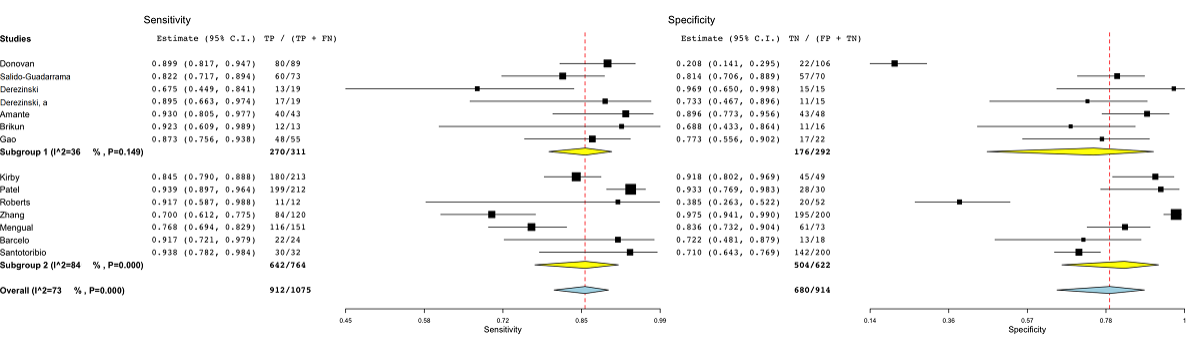
Subgroup analysis for the imbalance covariate in genomic studies. Subgroup 1: balanced data sets; subgroup 2: unbalanced data sets. FN: false-negative; FP: false-positive; TN: true-negative; TP: true-positive.

Finally, the effect of using balanced or highly unbalanced data sets in ML approaches was investigated (Figure 12). Seven studies were included in subgroup 2, as they employed highly unbalanced data sets. The heterogeneity of subgroup 1 was around 36%, whereas subgroup 2 showed a high heterogeneity (*I*^2^=84%, *P*<.001).

As a result, among several covariates, the imbalance covariate was the only one by which the heterogeneity could be partially resolved for more than 5 studies.

By inspecting Figure 12, Donovan et al [62] presented a very low value for specificity; this was due to the fact that they fixed the sensitivity threshold value at 90%.

**Figure 13:**
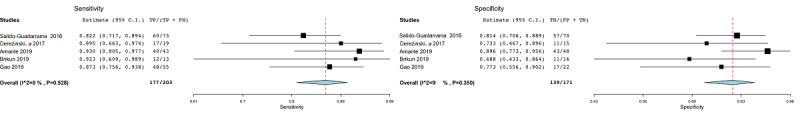
Coupled forest plots for balanced studies. The included studies investigated urine specimens. FN: false-negative; FP: false-positive; TN: true-negative; TP: true-positive.

Five studies employing urine specimens and balanced data sets showed a very low heterogeneity (Figure 13) [15,17,66,67,69].

**Figure 14:**
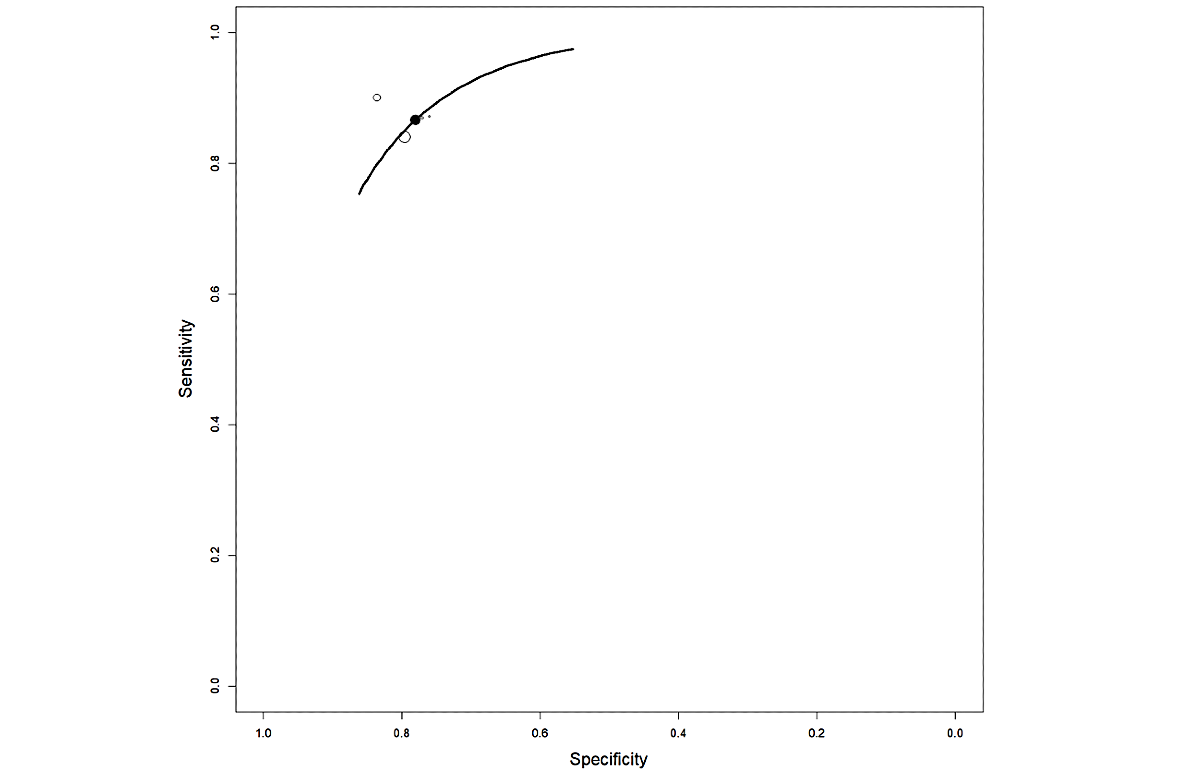
Hierarchical summary receiver operating characteristic curve (HSROC) for a subset of genomic studies. HSROC was calculated for genomic studies with low heterogeneity [15,17,66,67,69].

The HSROC curve for the studies employing balanced data sets to automatically detect PCa via urine biomarkers is shown in Figure 14. The pooled sensitivity and specificity were 0.812 (95% CI 0.577-0.999) and 0.8101 (95% CI 0.544-0.999), respectively.

## Discussion

### Principal Findings

This paper presents the results of a systematic literature review with meta-analysis of articles investigating machine learning algorithms to detect PCa via radiomic or genomic analysis. One research focus of this study was on clearly evaluating how the implementation of different ML approaches impacts the clinical results. At this stage, due to the high heterogeneity of methods and tools employed in the existing literature, no clear clinical relevance on the use of ML for PCa can be drawn from this study. This review shows that ML has helped to improve the diagnostic performance of the detection of PCa, but challenges still remain for clinical applicability of such methods, and more research is needed. The presented literature aims to help in building an ML system that is robust and computationally efficient to assist clinicians in the diagnosis of PCa via radiomic and genomic biomarkers.

In this review, 37 studies were shortlisted, and 29 studies were included in a meta-analysis. All patients were diagnosed with PCa by biopsy. However, not all the included studies reported full information on the methods used to carry out biopsy (eg, direct MRI-guided, cognitive fusion, or MRI-TRUS fusion biopsy).

In the radiomic and genomic meta-analysis, 16 and 14 studies were included, respectively. Heterogeneity among radiomic and genomic studies was 84% and 73%, respectively. This was expected, as ML methods are usually regarded as black boxes, and the consideration of all possible transformations is onerous. Moreover, there are no clear guidelines on how to develop AI approaches for medical studies, even though a few recommendations have been summarized by Foster et al [24] and Chen et al [25]. Another font of heterogeneity in radiomic studies may be due to the inclusion of PI-RADS score 3 and Gleason score 3+3 lesions, which are equivocal and should be disregarded in classification processes.

To partially solve the heterogeneity for the included studies, subgroup analyses were conducted based on several covariates. In the field of ML, applications where repeated measures or records have been captured on each subject can affect the overall performance. In most studies, the main aim is to predict if a given subject is “sick” or a “control” subject. In these applications, each subject has a single label type (eg, “sick” or control case). Nonetheless, there are other classification problems where each subject can have multiple labels. For instance, multiple lesions can be extracted from the same subject, and the control part can be represented by the benign-adjacent prostate lesion. It has been demonstrated that this phenomenon, known as identity confounding, can cause discrepancy in classification performance [70,71]. Therefore, the studies included in the meta-analysis were investigated to determine whether they explored patient- or lesion-based models. A patient-based model could be defined as a model that is developed and assessed in a “subject-wise” fashion, where all the records of each subject are considered as a group in the training and testing set and when assessing the model performance; conversely, a lesion-based model could be defined as a model that is developed and assessed in a “record-wise” fashion, where each measurement or record contributes to both the training and test sets and when assessing the model performance [70].

In both radiomic and genomic studies, patient-based models presented lower heterogeneity and performance than lesion-based models; this could be due to the fact that lesion-based models employed a bigger size sample, but the models may be overfit due to repeated measures.

A second important covariate to examine in ML problems is the data set construction. In particular, the data set is usually divided into training and testing sets in order to reduce overfitting problems [70,71]. The training set is often further split into a training set and a validation set, which is used to update model parameters. At least one procedure of internal or external validation is required in ML approaches. Cross-validation techniques are preferred if availability of data is not a problem. It is also strongly suggested to retrain on a subset of data or use an independent data set for external testing. Therefore, “validation approach” was used as a covariate in subgroup analysis. Validation approaches were divided into cross-validation, hold-out approach (split) or external validation, and no validation. In both radiomic and genomic analysis, studies employing cross-validation techniques and hold-out approaches had very high heterogeneity and similar performances among them. High heterogeneity may be due to the different cross-validation techniques used (eg, bootstrapping [16,40,52], Monte Carlo cross-validation [17]) or the choice of number of folders used in cross-validation methods; if an external data set was used [52,60,61,63], differences in the study protocols may have increased the bias among studies. Moreover, few studies in radiomic [50,53,57,59] and genomic [17,67] analysis employed both cross-validation and external testing. Studies employing no validation showed very low heterogeneity (only 2 studies in radiomic analysis), which may be due to the absence of other confounding variables, and high performances may be due to overfitting problems. A lower specificity was only noted in genomic analysis; this was due to Donovan et al [62], which used a fixed threshold for sensitivity at 90%.

Different ML approaches were also investigated among radiomic studies as a possible covariate factor. There were no relevant differences in heterogeneity or performance among subgroups (Figure 4). All genomic studies employed regression-based models. In fact, one limitation of the genomic studies was that none of the selected studies explored the potential of ML techniques at full capacity. Subgroup analysis was also conducted among radiomic studies employing ML or DL (ie, based on artificial neural networks) approaches. As expected, heterogeneity among DL studies was higher than among the studies employing other ML approaches to detect PCa. This could be mainly due to the high complexity of DL methods and hyperparameters. Moreover, DL approaches showed lower performance due to the small sample sizes used; they need large volumes of data to automatically identify patterns and achieve high performance.

The imbalance covariate was crucial in this study. Unbalanced and small data sets are very common in the medical field, and ML algorithms tend to produce unsatisfactory classifiers when handled with imbalanced data sets. Therefore, several techniques to overcome this problem have been proposed over time [72]. In this review, none of the studies included in the subgroup of unbalanced data sets had used any techniques to overcome the problem. Only one study [56] used SMOTE, but it did not employ a highly unbalanced data set.

For radiomic studies, after excluding studies that employed highly unbalanced data sets, the heterogeneity was less than 50%. The final pooled sensitivity and specificity for the use of mpMRI were 0.808 (95% CI 0.38-0.999) and 0.831 (95% CI 0.41-0.999), respectively.

For genomic studies, the heterogeneity dropped to 36% and reached a value close to zero when Donovan et al [62] was excluded because they fixed a threshold of 90% for sensitivity. The final pooled sensitivity and specificity were 0.812 (95% CI 0.577-0.999) and 0.8101 (95% CI 0.544-0.999), respectively. The predictor used to estimate the final pooled sensitivity and specificity was urine specimen.

Only 4 studies [18,39-41] investigating clinically based models were identified through the search. All the included studies adopted internal validation techniques (3 cross-validation [39-41] and 1 internal split validation [18]). Two studies [40,41] employed regression-based models, one [39] employed a tree-based model, and lastly, one employed a DL approach [18]. Heterogeneity was very high among them (*I*^2^=96%, *P*=.01) due to different sample sizes and diversity of predictors. However, contributions from genomic and imaging biomarkers should be considered to improve the overall performance of the clinically based diagnostic models.

Comparison among genomic and radiomic studies was not possible because they describe two different but complementary prospective approaches to the disease. However, the pooled sensitivity and specificity for both mpMRI and urine biomarkers were around 80%, showing them to be promising biomarkers in the detection of PCa via ML in clinical practice. The use of mpMRI has shown great diagnostic potential [73]; however, its analysis and interpretation are quite challenging, and there is not a consensus on how to optimally extract significant information. On the other side, genomic analyses have significantly increased our understanding of PCa and greatly improved patient risk classification, thus impacting treatment decision making. Therefore, a new prospective approach is the integration of radiomic and genomic signatures, commonly known as radiogenomics [74-76], in order to improve the overall performance of diagnostic tools to automatically detect PCa. In the existing literature, only a few studies have investigated “radiophenotypes” to complement existing validated clinical and genomic risk stratification biomarkers [77-79].

In this scenario, a typical ML postprocessing pipeline for radiomic and genomic analysis to automatically detect PCa may be constituted of a few crucial steps. In the case of radiomic studies, a common pipeline may be constituted of (1) examination of mpMRI; (2) image segmentation through the delineation of ROIs or VOIs, which can include whole gland volume, a specific zone, and one or multiple lesions, which should be explicitly specified in the manuscript; (3) image preprocessing; (4) filtering; (5) feature extraction; (6) integration of radiomic data with clinical data, genomic data, or both; (7) feature selection in relation to the target class; and (8) algorithm training, validation, and testing. Alternatively, a DL approach would only require the examination of the images and annotation of the ROIs or VOIs of the whole image, according to the desired classification output.

The image processing pipeline should be carefully described in the manuscripts, and the spatial coregistration of DWIs is a critical factor in the correct analysis of diffusion tensor imaging data, which has often been used as a predictor of PCa diagnosis. Moreover, the use of endorectal coil can cause high deformation of the prostate compared with other coils and may not provide adequate MR image quality [80]. Therefore, further processing of the images should also be considered, especially when the study is multicenter and different protocols have been adopted.

Due to the high heterogeneity of genomic studies, a standard pipeline configuration could be structured into (1) missing value management; (2) filtering to remove low-variance features; (3) data normalization due to data coming from heterogeneous formats; (4) a feature selection step to remove irrelevant features due to the high dimension of data; (5) dealing with class imbalance distribution present in this type of large-scale data set; and (6) algorithm training, validation, and testing. Alternatively, a DL approach would handle filtering and feature selection to generate handcrafted features. Deep learning is a powerful tool to integrate different “omics” and increase the computational power of diagnostic tools.

Further general recommendations on how to avoid bias and pitfalls in applying ML to medical problems are as follows: (1) in the case of multicenter studies, it is recommended to use batch effect approaches to prevent any bias due to different study protocols and feature normalization procedures to reduce within-subject bias [81]; and (2) for classifier performance, it is necessary to report if any threshold has been used to identify sensitivity and specificity and whether the performance was reported on patient-based or lesion-based data sets.

### Limitations

Our study presents several limitations. Some variability still remains due to the actual thresholds between studies. However, the multiple hierarchical model accounts for between- and within-subject variability among studies, including threshold effects. Another factor that could have affected the heterogeneity among studies is the use of different predictors among radiomic and genomic studies. Moreover, several studies reported little or incomplete information on the parameters used to develop ML models. Therefore, the number of parameters that are estimated by each technique was not investigated as a possible source of heterogeneity among studies. Additional heterogeneity in the observed results is due to the variability of calibration differences between equipment and differences between readers or observers, as well as variation in the implementation of tests. Another possible bias may be due to the preprocessing techniques on the extracted data and feature selection and feature normalization methods.

We limited the search to English-only studies; although this is common in systematic reviews, this exclusion criterion could have reduced the generalizability of the findings. However, the extent and effects of language bias have recently diminished because of a shift toward publication of studies in English [82]. At this stage, we also excluded PCa risk stratification studies to reduce bias and heterogeneity among studies, but further investigation on the use of ML methods to assess risk stratification biomarkers could give a comparative perspective on the treatment selection.

Finally, publication bias was not assessed in our analysis, as there are currently no statistically adequate models in the field of meta-analysis of diagnostic test accuracy [29].

### Conclusion

ML has shown its potential to empower clinicians in the detection of prostate cancer. The accuracy of ML algorithms for diagnosis of PCa was considered acceptable, in terms of heterogeneity, for 12 radiomic studies investigating mpMRI and 5 genomic studies using urine biomarkers.

However, given the limitations indicated in our study, further well-designed studies are warranted to extend the potential use of ML algorithms to clinical settings. Recommendations on the use of these techniques were also provided to help researchers to design robust studies aiming to identify radiomic and genomic biomarkers to detect cancer.

## Appendix

Multimedia Appendix 1 Supplementary material.

## Abbreviations

AI: artificial intelligence
AUC: area under the curve
DCE: dynamic contrast-enhanced
DL: deep learning
DWI: diffusion-weighted imaging
FN: false-negative
FP: false-positive
HSROC: hierarchical summary receiver operating characteristic curve
ML: machine learning
mpMRI: multiparametric magnetic resonance imaging
PCa: prostate cancer
PI-RADS-V2: Prostate Imaging Reporting and Data System
PRISMA: Preferred Reporting Items for Systematic Reviews and Meta-Analyses
PSA: prostate-specific antigen
QUADAS-2: quality assessment of diagnostic accuracy studies–version 2
ROI: region of interest
SMOTE: synthetic minority oversampling technique
TN: true-negative
TP: true-positive
TRUS: transrectal ultrasonography
TZ: transition zone
VOI: volume of interest
