# Supplementary material for: Radiomic and Genomic Machine Learning Method Performance for Prostate Cancer Diagnosis: Systematic Literature Review
Source: J Med Internet Res. 2021 Apr 1;23(4):e22394. doi: 10.2196/22394 (PMC8050752; doi:10.2196/22394)
Supplement: Multimedia Appendix 1 [file jmir_v23i4e22394_app1.docx]

**Supplementary material**

**Machine learning for prostate cancer diagnosis: a systematic literature review of radiomic and genomic method performance.**

**Contents:**

Supplementary Table S1. Search strings.

Supplementary Table S2. Inclusion and exclusion criteria.

Data extraction

Quality Assessment and Supplementary Fig. S1.

Supplementary Fig. S2. HSROC for all radiomic studies. Summary ROC based on hierarchical modelling.

Supplementary Fig. S3. Coupled forest plot for all the identified radiomic studies. The coupled forest plot was generated for all the radiomic studies included in the meta-analysis.

Supplementary Fig. S4. HSROC for all genomic studies. Summary ROC based on hierarchical modelling

Supplementary Fig. S5. Coupled forest plot for all the identified genomic studies. The coupled forest plot was generated for all the genomic studies included in the meta-analysis.

Supplementary Table S3. Overview of the primary datasets used in included studies.

Supplementary Table S4. Overview of modelling techniques.

Supplementary Table S5. PRISMA Checklist.

**Supplementary Table S1: search strings and results**

| **Database: PUBMED** | | | |
| --- | --- | --- | --- |
| **N** | **Keywords** | **Strings** | **Results (N)** |
| #1 | Prostate cancer | prostate cancer [Title/Abstract] | [112670](https://www.ncbi.nlm.nih.gov/pubmed/?cmd=HistorySearch&querykey=1) |
| #2 | Machine learning and synonymous | ((((((((((((((("artificial intelligence"[Title/Abstract]) OR "machine learning"[Title/Abstract]) OR "deep learning"[Title/Abstract]) OR "neural networks"[Title/Abstract]) OR "reinforcement learning"[Title/Abstract]) OR "naive bayes"[Title/Abstract]) OR "decision tree"[Title/Abstract]) OR "random forest"[Title/Abstract]) OR "support vector machine"[Title/Abstract]) OR "k nearest neighbor"[Title/Abstract]) OR "linear discriminant analysis"[Title/Abstract]) OR "regression"[Title/Abstract]) OR "classification"[Title/Abstract]) OR "clustering"[Title/Abstract]) OR "supervised learning"[Title/Abstract]) OR "unsupervised learning"[Title/Abstract] | [1116519](https://www.ncbi.nlm.nih.gov/pubmed/?cmd=HistorySearch&querykey=6) |
| #3 | Performance and synonymous | ((((((((("sensitivity"[Title/Abstract]) OR "specificity"[Title/Abstract]) OR "area under the curve"[Title/Abstract]) OR "area under curve"[Title/Abstract]) OR "positive predictive value"[Title/Abstract]) OR "negative predictive value"[Title/Abstract]) OR "true positive"[Title/Abstract]) OR "false positive"[Title/Abstract]) OR "true negative"[Title/Abstract]) OR "false negative"[Title/Abstract] | [1105715](https://www.ncbi.nlm.nih.gov/pubmed/?cmd=HistorySearch&querykey=3) |
| #4 | MRI, clinical data, biomarker | (((((((("mri"[Title/Abstract]) OR "magnetic resonance imaging"[Title/Abstract]) OR "imaging"[Title/Abstract]) OR "radiomic"[Title/Abstract]) OR "multiparametric magnetic resonance imaging"[Title/Abstract]) OR "mpmri"[Title/Abstract]))) OR ((("clinical data"[Title/Abstract]) OR "biomarker"[Title/Abstract])) | [1078522](https://www.ncbi.nlm.nih.gov/pubmed/?cmd=HistorySearch&querykey=4) |
| #1 AND #2 AND #3 AND #4 | | | 483 |
| #1 AND #2 And #3 and #4 from 2015-2020, English, Full Text | | | 240 |
| **Database: SCOPUS** | | | |
| #1 | Prostate cancer | TITLE-ABS (prostate AND cancer ) | 148,393 |
| #2 | Machine learning and synonymous | TITLE-ABS ( artificial AND intelligence ) OR TITLE-ABS ( machine AND learning ) OR TITLE-ABS ( deep AND learning ) OR TITLE-ABS ( neural AND networks ) OR TITLE-ABS ( reinforcement AND learning ) OR TITLE-ABS ( naive AND bayes ) OR TITLE-ABS ( decision AND tree ) OR TITLE-ABS ( random AND forest ) OR TITLE-ABS ( support AND vector AND machine ) OR TITLE-ABS ( k AND nearest AND neighbor ) OR TITLE-ABS ( linear AND discriminant AND analysis ) OR TITLE-ABS ( regression ) OR TITLE-ABS ( classification ) OR TITLE-ABS ( clustering ) OR TITLE-ABS ( supervised AND learning ) OR TITLE-ABS ( unsupervised AND learning ) | 2,901,350 |
| #3 | Performance and synonymous | TITLE-ABS ( sensitivity ) OR TITLE-ABS ( specificity ) OR TITLE-ABS ( area AND under AND the AND curve ) OR TITLE-ABS ( area AND under AND curve )OR TITLE-ABS ( positive AND predictive AND value ) OR TITLE-ABS ( negative AND predictive AND value ) OR TITLE-ABS ( true AND positive ) OR TITLE-ABS ( false AND positive ) OR TITLE-ABS ( true AND negative ) OR TITLE-ABS ( false AND negative ) | 2,092,285 |
| #4 | MRI, clinical data, biomarker | TITLE-ABS ( mri ) OR TITLE-ABS ( magnetic AND resonance AND imaging ) OR TITLE-ABS ( imaging ) OR TITLE-ABS ( radiomic ) OR TITLE-ABS ( multiparametric AND magnetic AND resonance AND imaging ) OR TITLE-ABS ( mpmri ) OR TITLE-ABS ( clinical AND data ) OR TITLE-ABS ( biomarker ) | 2,410,847 |
| #1 AND #2 AND #3 AND #4 | | | 1,002 |
| #1 AND #2 AND #3 AND #4 from 2015-2020, English, Full Text | | | 454 |
| **Database: OVID^SP^** | | | |
| #1 | Prostate cancer | Prostate cancer.ab,ti. | 285923 |
| #2 | Machine learning and synonymous | (artificial intelligence or machine learning or deep learning or neural networks or reinforcement learning or naive bayes or decision tree or random forest or support vector machine or k nearest neighbor or linear discriminant analysis or regression or classification or clustering or supervised learning or unsupervised learning).ab,ti. | 3021460 |
| #3 | Performance and synonymous | (sensitivity or specificity or area under the curve or positive predictive value or negative predictive value or true positive or true negative or false positive or false negative or area under curve).ab,ti. | 2717125 |
| #4 | MRI, clinical data, biomarker | (mri or magnetic resonance imaging or imaging or radiomic or multiparametric magnetic resonance imaging or mpMRI or clinical data or biomarker).ab,ti. | 2751408 |
| #1 AND #2 AND #3 AND #4 | | | 3485 |
| #1 AND #2 AND #3 AND #4 from 2015 to 2020, full text, English | | | 183 |

**Supplementary Table S2: Inclusion and Exclusion Criteria**

| **Inclusion Criteria:** |
| --- |
| 1. the object of the study was to differentiate individuals and/or lesions clinically diagnosed with Prostate Cancer (PCa) from controls (e.g., healthy subjects, non-cancerous lesions); |
| 1. algorithms, suggested as machine learning in the study, including appropriate apparatus, such as learning or training, aimed at automatically detecting PCa; |
| 1. MRI or genomic biomarkers and/or clinical data were used as predictors in ML algorithms; and, |
| 1. articles with stated or easily derived true-positive (TP), true-negative (TN), false-positive (FP), and false-negative (FN) rates, sensitivity and specificity. |
| **Exclusion Criteria:** |
| 1. all review articles, letters, abstract, conference paper and case reports; |
| 1. the study only included individuals at high risk for PCa and/or without diagnostic confirmation; |
| 1. studies featuring patients with previous recurrences and those who have received radiation therapy for PCa; |
| 1. training, learning, and/or validation process were not explained clearly or distinguished from each other; |
| 1. non-human subjects (e.g., animals); and, |
| 1. non-English papers. |

**Supplementary material: detailed process of data extraction**

The data extraction items could be broadly grouped into three areas (A1 to A3): A1) basic information of individual studies and participations (or dataset), A2) performance and validation condition, and A3) accuracy indices.

For A1 (basic information of individual studies domain), the name of the first author, publication year and type were extracted. Timing of the studies (retrospective, prospective, cohort, cross-sectional, case control) was also recorded. Dataset domain was divided into training dataset and validation dataset. Validation dataset can be further split in different types: internal validation, whose sample originates from the same sample as the training dataset, and external validation, whose samples are from an independent sample data. Internal validation can be further split in internal-split validation, which uses a sample that has been removed from the original dataset for the purpose of testing, and internal-cross validation, which repeats validation process over a sample that is removed from the training dataset. For machine learning algorithms, there are two types of dataset: 1) public – open to anyone with anonymized sensitive information, 2) private – requires permission from an organization, institution, or a hospital. For sample size, the number of target condition (PCa positive and negative) and the number of extracted data per participant (e.g., the number of MRI images, lesions or tissue samples) were extracted separately, in order to see if input data had been extracted repeatedly from a single participant. Participants’ average age, Gleason score and PSA levels were also extracted. Whether individuals with PCa or certain subtype(s) of PCa were included was assessed and used in quality assessment of applicability concern for patient selection. Last, indicative parameters of dataset quality were extracted. Machine learning algorithms (in particular, imaging studies) are known to be influenced by the quality of input data. Quality parameters, such as device resolution - e.g., slice thickness in imaging studies, presence or absence of calibration in biochemical marker studies – were extracted.

For A2 (performance and validation condition domain) acquisition or pre-processing analyses, during which input data are simply processed were recorded. Second, for segmentation, by which input data are broken into smaller regions, whether segmentation was performed or not, as well as, whether machine learning algorithms were used or not were considered. Third, detailed methods of input data for machine learning algorithms were extracted. Fourth, the type of predictors and algorithms were extracted.

For A3 (accuracy indices domain), all accuracy values were extracted, in particular true-positive/true-negative/false-positive/false-negative (TP/TN/FP/FN) values for meta-analysis. Moreover, the used attributes (extracted features) were not important as the types of artificial intelligent systems were different and were not considered for the meta-analysis approach.

### Supplementary material: Quality assessment

The QUADAS-2 tool comprises of 4 sections: “patient selection,” “reference standard”, “index test” and “flow and timing”. QUADAS-2 is a validated tool used to evaluate the quality of diagnostic accuracy studies and risk of bias (RoB) for internal and external validity of individual studies. Two authors (RC and MF) independently assessed the quality of individual studies. There was no disagreement between authors. In this study, the index test is a machine learning algorithm. The machine learning algorithm’s accuracy is reported through a process called validation. However, when a study provided no information about the validation process, input parameters and sample size, high RoB was assumed. Otherwise, the level of RoB was determined by thoroughly reviewing the validation process.

Of the 37 studies included in the qualitive assessment, six, nine and two studies were assessed to have high RoB by flow and timing, machine learning approaches and patient selection, respectively. Only one study was assed to have an unclear RoB in reporting machine learning approach. For applicability concerns, only four studies validated their models on an external dataset. All of them showed to have a low RoB. The distribution of the RoB for the included studies is represented in Supplementary Fig. S1.


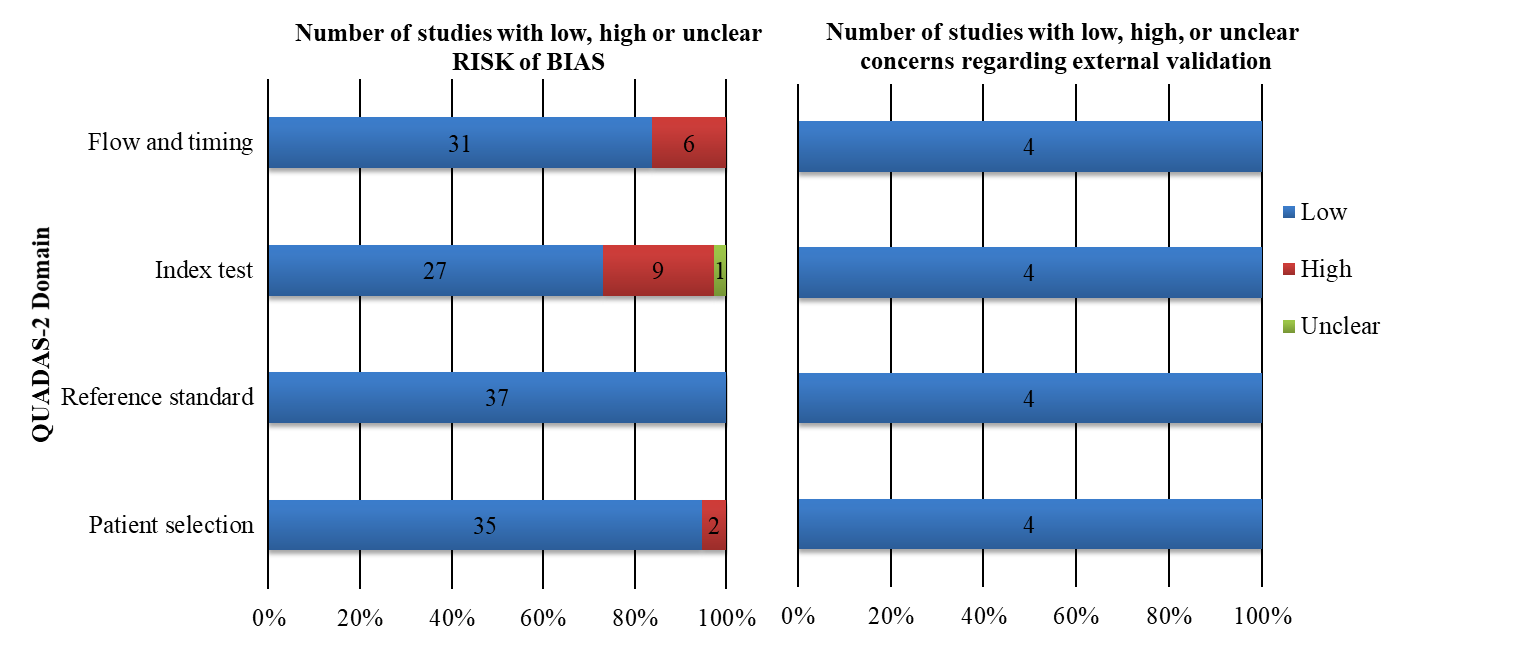


**Supplementary** **Fig. S1 Distribution of the RoB for the included studies.** Risk of bias (RoB) and applicability concern by domain in Quality Assessment of Diagnostic Accuracy Studies-2.

**Supplementary Fig. S2**


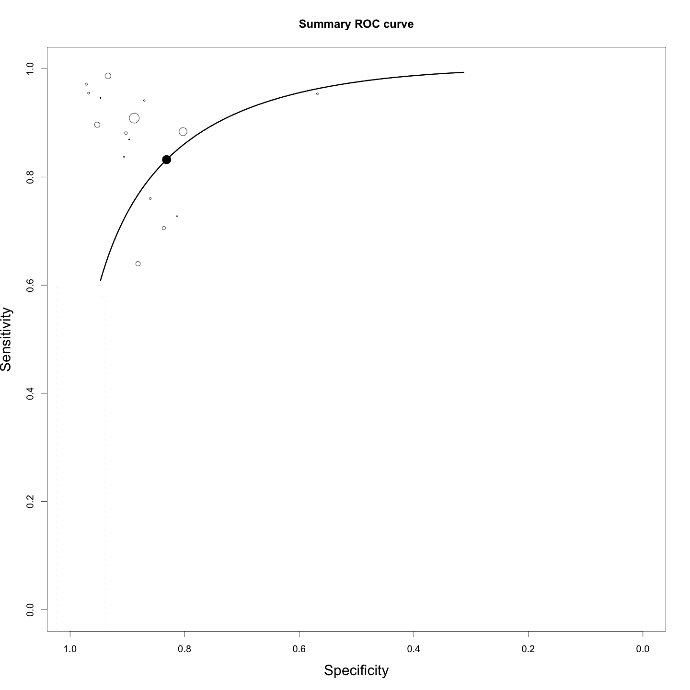


**Supplementary Fig. S2** **HSROC for all radiomic studies.** Summary ROC based on hierarchical modelling.

**Supplementary Fig. S3**


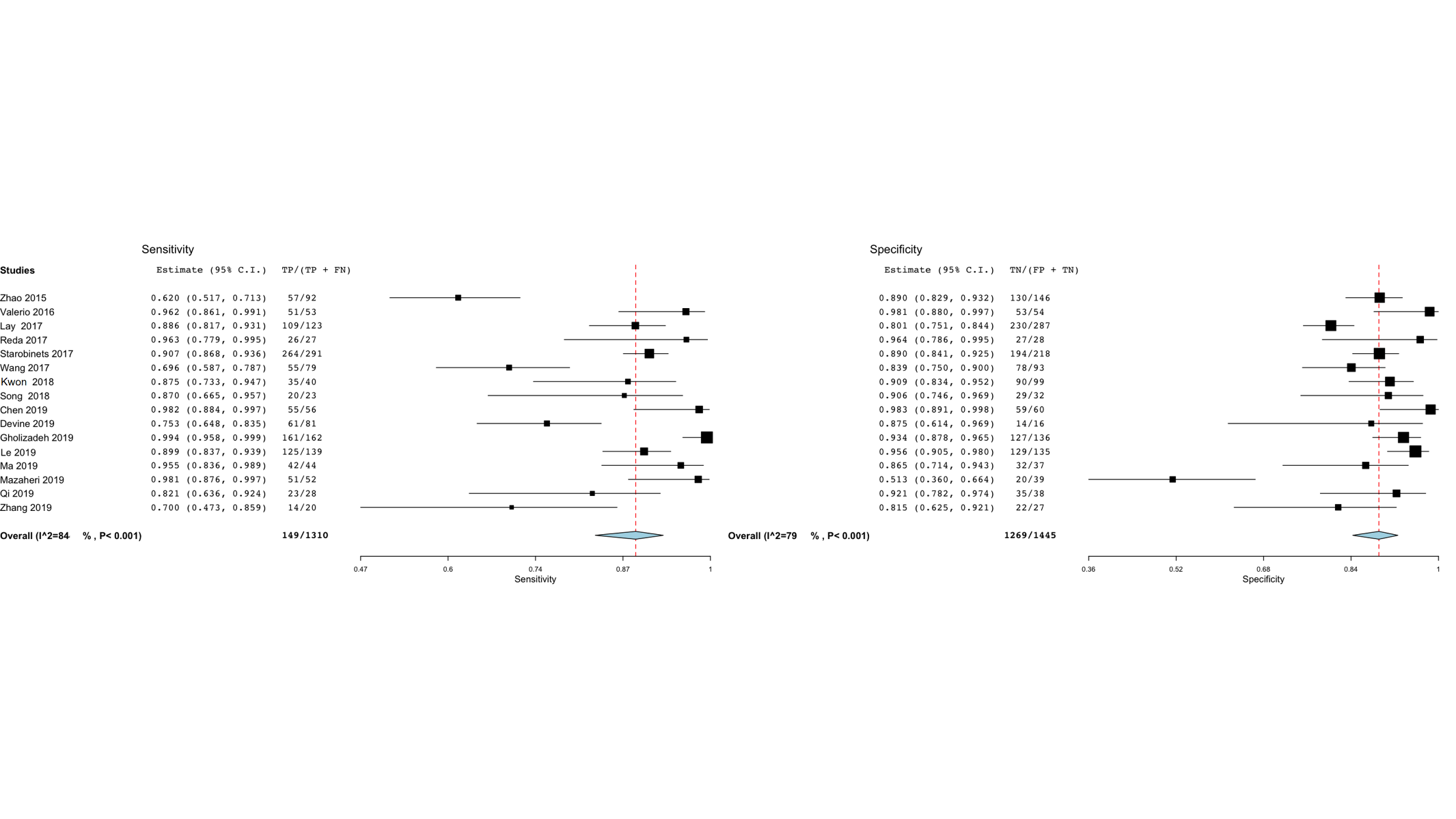


**Supplementary Fig. S3 Coupled forest plot for all the identified radiomic studies**. The coupled forest plot was generated for all the radiomic studies included in the meta-analysis.

**Supplementary Fig. S4**


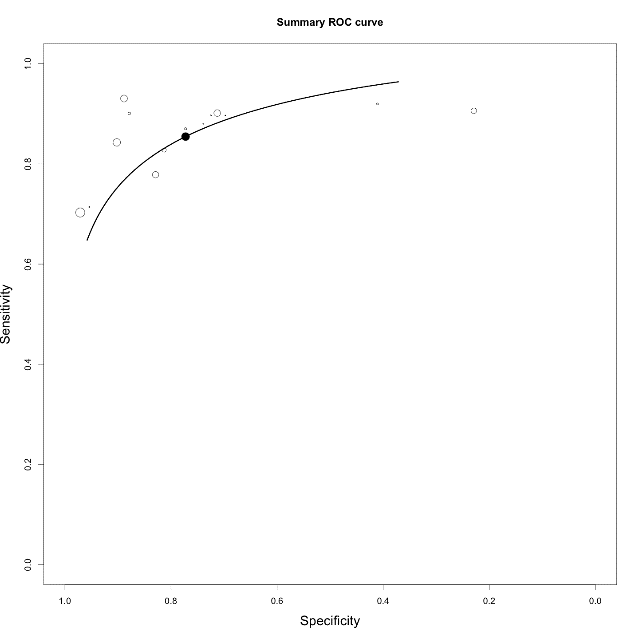


**Supplementary Fig. S4 HSROC for all genomic studies.** Summary ROC based on hierarchical modelling

**Supplementary Fig. S5**


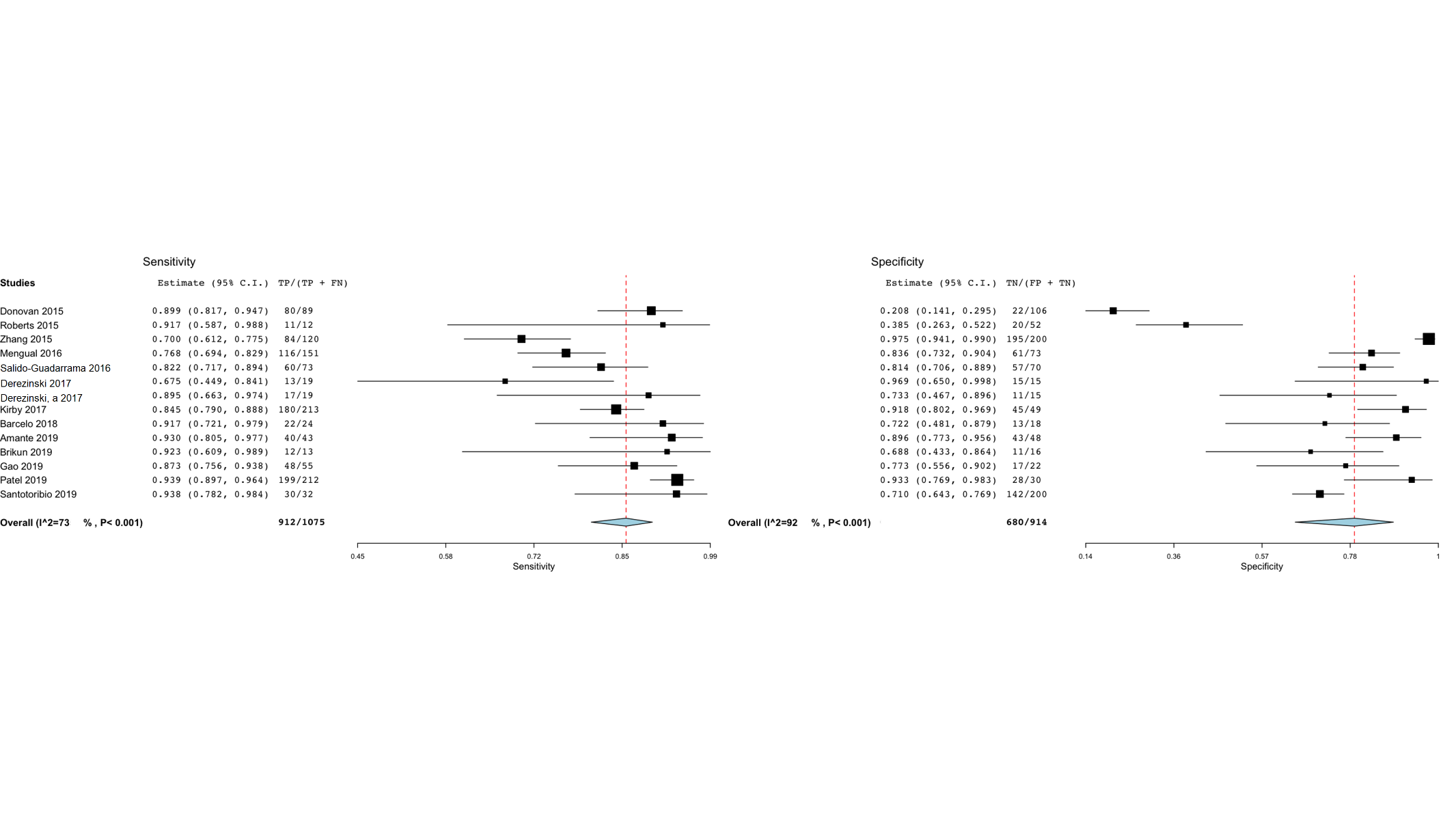


**Supplementary Fig. S5 Coupled forest plot for all the identified genomic studies.** The coupled forest plot was generated for all the genomic studies included in the meta-analysis.

**Supplementary Table S3. General characteristics of the studies included in the systematic review.**

| **Author, Year** | **Timing (retrospective, prospective, cohort, cross-sectional, case control)** | **Dataset Character** | | **Number of Sample,**  **n (=positive PCa +, negative Pca - )** | | | **Characteristics of sample (PCa +, -)** | | | **Disease characteristics** | **Disease.**  **position**  **(TZ,**  **PZ, CG)** | **Data quality parameters** | **Accuracy Indices** |
| --- | --- | --- | --- | --- | --- | --- | --- | --- | --- | --- | --- | --- | --- |
|  |  | **Subset** | **Type (name)** | **Per Patients (+, -)** | **Per cases (images, genes, etc)** | **Patients/ images/lesion/tissue samples** | **Age: Mean (SD) or Mean [Range]** | **Gleason Score (n, %)** | **PSA (ng/ml ): Mean (SD) or Mean [range] or count** |  |  |  |  |
| **Radiomic Studies** |  |  |  |  |  |  |  |  |  |  |  |  |  |
| Chen, 2019 | Retrospective study | internal-split validation | Private (Hospital) | 381(182, 199) | 381 (182, 199) | patients-based model | Pca:74 [56–90]; non-PCA: 68 [55–88] | GS 6: 40 (22%); — GS 7: 62 (34%); —  GS 8: 38 (21%); —  GS 9: 32 (18%); —  GS 10:10 (5%) | PSA≤10: 31 PCa, 109 non-PCa. 10<PSA≤20: 34 PCa, 57 non-PCa. PSA>20: 117 PCa, 33 non-PCa. | PCa and Healthy | PZ | All scans were performed using a 3.0T MRscanner (Philips Intera Achieva, Best, Netherlands) with a 32-channel body phased array coil as the receiving coil. Scan sequences included sagittal T2WI, axial T2WI, T1WI, DWI (b values of 0 and 1000 sec/mm2) and DCE. | Sensitivity, specificity and overall accuracy |
| Devine, 2019 | Prospective study | internal cross-validation | Private (Hospital) | 65 | 97(81, 16) | lesion-based model | 65 [49–79] | GS 6: 3; —GS 7:9; —GS 8: 2;— GS 9: 1 | N/A | PCa | PZ | Subjects were scanned on a 3.0 T scanner (Philips Achieva; Philips Medical Systems, Best, The Netherlands) using a 32‐channel cardiac coil. A multiecho spin‐echo sequence with an echo spacing of 31.25 msec and repetition time (TR) of 8956 msec was used. The other parameters were: NE = 32; field of view (FOV) = 180 × 180 mm; acquired voxel size = 2 × 2 × 4 mm; scan duration = 5 minutes 50 seconds. DWI was acquired for VERDICT fitting with single diffusion encoding (SDE) single‐shot EPI sequences over six b‐values (0, 90, 500, 1500, 2000, 3000 s/mm2). TR/TE = 2000–3707/50–80 msec; FOV = 220 × 220 mm; voxel size = 1.3 × 1.3 × 5 mm; scan duration = 12:57. | Sensitivity, Specificity and AUC. |
| Gholizadeh, 2019 | Retrospective study | internal cross-validation | Private (Hospital) | 11 | 297(161, 131) | lesion-based model | 71.4 (9.2) | GS: 4+3:6;— GS: 4+5:5 | 16.6(12.9) | PCa | PZ | The MR images were obtained with a 3 T Skyra scanner (Siemens Healthineers, Erlangen, Germany, maximum gradient amplitude (single axis): 45 m T/m, minimum gradient rise time: 225 μs, maximum gra- dient slew rate: 200 T/m/sec) using an external 18-channel phase array coil (Siemens Healthineers, Erlangen, Germany). A two-dimensional turbo spin echo (TSE) T2WI of the prostate was performed axially with the following parameters: repetition time (TR): 1400ms; echo time (TE): 96 ms; number of averages: 3; flip angle: 135°; slice thickness: 4 mm; no gap; field of view (FOV): 200 mm; and total acquisition time: 3.45 min. | sensitivity, specificity, Accuracy, AUC |
| Le, 2017 | Retrospective study | external validation | Private (Hospital) and TCIA dataset | 364(276, 88) | 913 (463,450) | lesion-based model | PCa: 67.5 (8.8); BPH: 66.5(8.6) | N/A | PCa:91.99(143.4); BPH:11.92 (14.18) | PCa and BPH | N/A | 3.0 T whole-body unit MR imaging system (MAGNETOM Skyra, Siemens Medical Solutions, Erlangen, Germany). An 18-element body matrix coil and the built-in 32-element spine matrix coil were used for signal reception. Twelve-core system- atic TRUS-guided plus targeted prostate biopsy was performed as part of the research protocol on all patients after the MRI examination. The acquisition parameters for obtaining the transverse, coronal and sagittal T2WI turbo spin-echo images were set as follows: repetition time (TR) 6750ms, echo time (TE) 104ms, echo train length 16, section thickness 3mm, no intersection gap, field of view (FOV) 180 mm × 180 mm, image matrix 384 × 384. The transverse plane of DWI sequences was obtained using a single-slot spin-echo echo-planar imaging sequence with two b values (0 and 1000s mm−2), TR/TE 4500ms/85ms, section thickness 3mm, FOV 214mm × 171mm and image matrix 90 × 72. The ADC maps were computed using Advanced Workstation. MRI protocols for data acquisition of the TCIA dataset are provided in Litjens et al. | sensitivity, specificity, accuracy, and AUC. |
| Kwon, 2018 | Retrospective study | internal cross-validation | SPIE-AAPM-NCI PROSTATEx challenge | 204 | 191 (36, 155). | lesion-based model. | N/A | N/A | N/A | PCa | PZ | The images were acquired on two different types of Siemens 3T MR scanners, the MAGNETOM Trio, and Skyra. 2-weighted images were acquired using a turbo spin echo sequence and had a resolution of around 0.5 mm in plane and a slice thickness of 3.6 mm. The DCE time series was acquired using a three-dimensional (3-D) turbo flash-gradient echo sequence with a resolution of around 1.5 mm in-plane, a slice thickness of 4 mm, and a temporal resolution of 3.5 s. The proton density weighted image was acquired prior to the DCE time series using the same sequence with different echo and repetition times and a different flip angle. Ktrans, the volume transfer coefficient that measures capil- lary permeability, computed from the DCE23 was provided in MetaImage medical format (mhd). Finally, the DWI series were acquired with a single-shot echo planar imaging sequence with a resolution of 2-mm in-plane and 3.6-mm slice thickness and with diffusion-encoding gradients in three directions. Three b-values were acquired (50, 400, and 800), and subsequently, the apparent diffusion coefficient (ADC) map was calculated by the scanner software. All images were acquired without an endorectal coil. | sensitivity, specificity, and area under the curve, PPV, NPV |
| Lay, 2017 | Retrospective study | internal cross-validation | Private (Hospital) | 224 | 410( 123, 287) | lesion-based model | N/A | GS 6: 23; —GS 7: 26; —GS 8: 12;— GS 9: 10; —GS 10: 3 | N/A | PCa | PZ | The mpMRI images were acquired using a 3T MR scanner (Achieva-TX, Philips Medical Systems, Best, the Netherlands) using the anterior half of a 32 channel SENSE cardiac coil (In vivo, Philips Healthcare, Gainesville, Florida) and an endorectal coil (ERC) (BPX-30, Medrad, Indianola, Pennsylvania). No pre-examination bowel preparation was required. The balloon of each ERC was distended with ∼45 ml of perfluorocarbon (Fluorinert FC-770, 3M, St. Paul, Minnesota) to reduce imaging artifacts related to air-induced susceptibility. The T2W image was acquired with an echo time, repetition time, and section thickness of 4434 ms, 120 ms, and 3 mm, respectively. The standard diffusion weighted imaging (DWI) was acquired with five evenly spaced b-values (0–750), and high b-value DWI was acquired with b 1⁄4 2000. | sensitivity, specificity, and AUC. |
| Ma, 2019 | Retrospective study | none | Private (Hospital) | 81(44, 37) | 81(44, 37) | Patient-based model | Pca:74 (7) ; BPH: 72 (11) | GS6: 12; — GS 8:11; — GS 9: 6; —GS 10: 7. | N/A | Pca and BPH | PZ and TZ | 3.0 Tesla MR scanner (Siemens Healthcare GmbH, Erlangen, Germany), equipped with a 16-channel body phased array surface coil. T2WI as follows: Repetition time (TR)=4,000 msec, echo time (TE)=96 msec, thickness=3 mm, field-of-view (FOV)=240x240 mm, number of excitations (NEX)=2, matrix=320x320. DWI scans were performed using a single shot echo sequence as follows: TR=4,500 msec, TE=93 msec, thickness=4 mm, FOV=260x221 mm, NEX=4, matrix=160x120. B values of 50 and 700 s/mm2 were selected, and ADC was reconstructed automatically after the scan. DCE-MRI used three-dimen- sional volumetric interpolated body scanning sequence of axial T1WI. Galodinium-diethylenetriamine penta-acetic acid (Gd-DTPA) contrast (Hokuriku Seiyaku Co., Ltd, Katsuyama, Japan) was administered intravenously via the median cubital vein at a dose of 0.1 mmol/kg and a rate of 2 ml/sec. After injection, the solution was flushed with 10 ml 0.9% sodium chloride. The protocol scanning parameters of DCE-MRI were as follows: TR=5.80 msec, TE=2.17 msec, thickness=3.60 mm, FOV=260x211 mm, matrix=352x352, NEX=1 and scanning repetitions=15. | sensitivity, specificity, accuracy, and AUC. |
| Mazaheri, 2019 | Retrospective study | internal cross-validation and split-validation | Private (Hospital) | 67 | 170 (102,58) | lesion-based model | 61.6 [41.5-74.7] | GS 6:11 (16.4);—GS>7: 56 (83.6) | 5.0 [0.1-65.8] | PCa | PZ | All MRI studies were performed on a 3T unit (Signa HDX; GE Healthcare, Milwaukee, Wis). The unit was equipped with gradient coil designed to achieve 50-mT/m peak gradient amplitude at a slew rate of 150 T/m/s. A body coil was used for excitation. For signal reception, both a pelvic eight-channel phased-array coil and a commercially available balloon-covered expandable endorectal coil (Medrad, Warrendale, Pa, USA),inflated with barium sulfate (E-Z-EM, Inc., Lake Success, NY) to about 60-100 mL, were used. For MRSI studies, only the endorectal coil was used for signal reception. MRSI data were acquired with the acquisition software (PROSE; General Electric Medical Systems, Milwaukee, WI) provided by GE under a research agreement, with a HIPAA-compliant, IRB-approved protocol. | sensitivity, specificity, accuracy, and AUC. |
| Qi, 2019 | Retrospective study | internal cross-validation and split-validation | Private (Hospital) | 199(85,114) | 199(85,114) | Patient-based model | Training PCa: 67.53 (7.28); Non-PCa: 62.51(10.05). Testing PCa: 66.39 (8.09), non-PCa: 61.82 (7.32) | Training: GS 6: 25 (43); —GS>6: 32(56). Testing: GS 6: 17 (60); —GS>6:11 (39) | Training PCa: 7.04 (1.67), non-PCa: 6.97(1.61). Testing PCa : 6.96(1.49), non-PCa:6.89(1.66) | PCa and Healthy | PZ | MRI was performed with a 3.0T MR scanner (Discovery MR 750, GE Medical Systems, Milwaukee, WI). The mpMRI protocol consisted of T2W, DW or DCE images. Refer to the paper for more information. | sensitivity , specificity, Accuracy, AUC |
| Reda, 2017 | Retrospective study | internal cross-validation | Private (Hospital) | 18 | 57 (26, 27) | lesion-based model | N/A | N/A | N/A | PCa | N/A | DW-MRI data sets, which have been acquired in the axial plane with a body-coil Signa Horizon GE scanner using the magnetic field strength of 1.5 Tesla; TE of 84.6 ms; TR of 8 s; bandwidth of 142.86 kHz; field of view of 34 cm; slice thickness of 3 mm with no inter-slice gap; conventional EPI acquisition sequence; mono-directional diffusion weighting, and range of b-values from 0 to 700 s/mm2. | sensitivity , specificity, Accuracy, AUC |
| Song, 2018 | Retrospective study | internal cross-validation and split-validation | SPIE-AAPM-NCI PROSTATEx challeng | 195 | 547 (261, 286) | lesion-based model | N/A | N/A | N/A | PCa | PZ | The axial T2WI, DWI (b-value of 50, 400, and 800 s/mm2) andADC maps were used for imaging analysis. All data were acquiredon 3T MR scanners with pelvic phased-array coils (MagnetomTrio and Skyra, Siemens Healthcare, Erlangen, Germany). AxialT2WIs were acquired using a turbo-spin-echo sequence with an in-plane resolution of 0.5 3 0.5 mm2and a slice sickness of 3.6mm. The DWI series was acquired with a single-shot echo-planar-imaging sequence, an in-plane resolution of 2 mm, and a slicesickness of 3.6 mm | The sensitivity, specificity, positive predictive value (PPV), and negative predictive value (NPV) |
| Starobinets, 2017 | Retrospective study | internal cross-validation | Private (Hospital) | 169 (102, 67); | 509 (291, 218) | lesion-based model | All: 63.7 (6.3) | ALL: GS> 6: 7 ([6- 8], | All: 6.2 [4.3 -8.35] | PCa and Healthy | PZ | All patients were imaged with an expandable balloon endorectal coil (MedRad, Bayer HealthCare LLC, Whippany, NJ, USA) combined with anexternal phased array coil on a 3 T MR scanner (GE Healthcare, Waukesha, WI, USA). A perfluorocarbon fluid (Galden; Solvay Plastics, WestDeptford, NJ, USA) was used to inflate the balloon coil. Fast spin echo (FSE) T2‐weighted images were acquired in an oblique axial plane withFOV = 18 cm, slice thickness = 3 mm, matrix = 512 × 512, and TR/TE= 6000/96 ms. DWI was acquired using a 2D single‐shot spin echo sequence,TR/TE= 4000/78–90 ms, pixel bandwidth = 1952 (conventional acquisition) or pixel bandwidth = 1305 (reduced‐field‐of‐view acquisition18), b =0and 600 s/mm2, slice thickness = 3 mm. Magnetic resonance spectroscopic imaging (MRSI) data was acquired using a 3D flyback, echo planarPRESS CSI acquisition, with a 16 × 12 × 10 matrix, acquired at 5.4 mm resolution, zero‐filled to 5.4 × 2.7 × 2.7 mm3, 0.04 cm3voxels, TR/TE= 2000/85 ms. | sensitivity (SN), specificity (SP), Accuracy, AUC |
| Valerio, 2016 | Retrospective study | none | Private (Hospital) | 53 | 106 (53,53) | lesion-based model | N/A | N/A | N/A | PCa | PZ | MR imaging of the pelvis was performed using a 3-T scan (Dis-covery MR750, GE Healthcare, Milwaukee, USA) equipped withan eight-channel torso phased-array coil and an endorectal coil. The MR imaging protocol of the prostate included the following sequences: - T2-weighted turbo spin echo sequences (repetition time (TR), 4500ms; echo time (TE), 110ms; thickness, 3mm; matrix, 352 × 352) acquired in the axial, sagittal and coronal planes. - Diffusion-weighted (DW) sequences (slice thickness, 3 mm; TR, 3100 ms; TE, 102 ms; exponential b values of 0, 500, 1000 and 3000 s/mm2 ) acquired in the axial plane. Average ADC (apparent diffusion coefficient) values were calcu- lated by placing a region of interest (ROI) on the suspicious areas on the ADC maps obtained. IVIM parameters were acquired with 11 b-values (0, 10, 20, 30, 40, 50, 80, 100, 200, 400, 800 s/mm2 ). - Dynamic contrast-enhanced MRI obtained using a gradient- echo T1-weighted sequence in the axial plane (TR, 3ms; TE, 2 ms; thickness, 3 mm; time resolution, 12 sections/3 s; matrix, 320 × 192). | sensitivity, specificity, AUC |
| Wang, 2017 | Prospective study | internal cross-validation | Private (Hospital) | 172 (79, 93) | 172 (79, 93) | Patient-based model | PCa:67.9(50–88); non-PCa: 66.5(47–91) | N/A | N/A | PCa and benign BPH + prostatitis | N/A | All MR images were performed by using a 3.0 Tesla (T) whole-body unit (MAGNETOM Skyra, Siemens Medical Solutions, Erlangen, Germany) followed by pathology reports and one year’s follow-up. The acquisition protocol included T2-weighted imaging (T2WI), T1-weighted imaging (T1WI), dynamic contrast-enhanced MRI (DCE-MRI) and diffusion-weighted imaging (DWI). The acquisition parameters for obtaining the 2dimensional transverse T2WI turbo spin-echo images were set as follows: repetition time (TR) 6750 ms, echo time (TE) 104 ms, echo train length 16, section thickness 3 mm, no intersection gap, field of view (FOV) 180 mm × 180 mm, image matrix 384 × 384. | Sensitivity, specificity, positive predictive value (PPV), negative predictive value (NPV), and AUC |
| Wu, 2019 | Retrospective study | internal cross-validation | Private (Hospital) | 90(46, 44) | 105(44, 61) | lesion-based model | 68 (7) | GS 3 + 4: 26; — GS 4 + 3: 12; — GS 9 or 10: 6 | N/A | PCa and BPH | TZ | Patients underwent MRI on one of two clinical 3 T systems (TRIO Tim, Siemens Healthcare, Erlangen, Germany, between May 2012 to December 2014 and Discovery 750 W, General Electric Healthcare, from May 2012 to the present). | Sensitivity, specificity, accuracy, and AUC. |
| Yang, 2017 | Prospective study | internal split-validation | Private (Hospital) | 160 (72, 88) | 160 (72, 88) | patient-based model | PCa: 67.5 (8.8); BPH: 66.5 (8.6) | GS 5 : 2; — GS 6: 17; — G S 7: 55; — GS 8: 38; — G S 9: 21 | Pca: 91.99(143.4) ; BPH: 11.92 (14.18 ) | Pca and BPH | TZ | All MR images used in this study were acquired on a 3.0 Tesla (T) whole-body unit MR imaging system (MAGNETOM Skyra, Siemens Medical Solutions, Erlangen, Germany), running software version Syngo MR D13. An 18-element body matrix coil and the built-in 32-element spine matrix coil were used for signal reception in the patients. | Sensitivity, specificity, and AUC |
| Zhang, 2019 | Retrospective study | internal cross-validation and split-validation | Private (Hospital) | 140(60, 80) | 140(60, 80) | Patient-based model | Training: 64.03 (10.09); Testing: 63.19 (7.39) | N/A | Training: 6.91 (1.64); Testing: 7.10 (1.58) | PCa and Healthy | PZ | The acquisition parameters of DWI data were as following: b values = 0 and 800s/mm2; repetition time/echo time = 3000 ms/56 ms; slice thickness = 6 mm; and acquisition matrix = 128*128. | Sensitivity, Specificity, NPV, PPV, Accuracy, AUC. |
| Zhao, 2015 | Prospective study | internal cross-validation | Private (Hospital) | 71 (35, 36) | 238 (92, 146) | lesion-based model | PCa: 68.8(8.9); non-PCa: 67.8(6.4) | N/A | PCa: 60.5(77.8) ; non-PCa:11.7 (8.1) | PCa and Healthy | PZ | All MR examinations were carried out on a 1.5 Tesla MR scanner (Signa TM; GE Medical Systems, Milwaukee, WI, USA) using a pelvic phased-array coil (GE Medical Sys- tems). All routine prostate MR examinations followed standardised protocols. T2-weighted turbo-spin-echo imag- es were obtained in the axial, sagittal, and coronal planes without fat suppression with TR: 3500 ms; TE: 85 ms; FOV: 240 mm×240 mm; matrix: 320×256; slice thickness: 4 mm with no gap; number of signals acquired: 4. | Sensitivity, Specificity, NPV, PPV, Accuracy, AUC. |
| Bonekamp, 2018 | Retrospective study | internal cross-validation and split-validation | Private (Hospital) | 316 | 462 (140, 322) | lesion-based model (VOI) | Training:64 [ 58–71]; testing: 63 [58–71] | Training: GS 6: 35 (19); —GS>6: 72(39). Testing: GS 6: 34 (25); —GS>6:49 (37) | Training: 6.6 [4.9–9.5]; Testing: 7.5 [5.4–11] | PCa | PZ | MR images at 3 T were acquired prior to biopsy according to the European Society of Urogenital Radiology, or ESUR, guidelines (Magnetom Prisma, Siemens Healthcare, Erlangen, Germany), by using the standard multichannel body coil and integrated spine phased-array coil. T2-weighted, DWI, and dy- namic contrast material2enhanced MR images were acquired according to the institutional prostate MRI protocol. ADC images, diffusion-weighted images with b value of 1500 sec/mm2, and T2-weighted images were ex- tracted and upsampled to 0.25- mm in-plane resolution and 3-mm section thickness by us- ing the medical imaging toolkit | sensitivity, specificity, accuracy, and area under the curve |
| Wang, 2017 | Retrospective study | internal cross-validation | Private (Hospital) | 54 | 101 (54, 47 ) | lesion-based model (VOI) | 74 [ 65.7–78.3] | GS 3 + 3: 14 (25.9); — GS 3 + 4: 11 (20.4);—  GS 4 + 3: 10 (18.5);—  GS 4 + 4 19 (35.2) | 23.6 [ 12.5–56.1] | PCa | PZ | All images were acquired with a 3.0-T MR scanners (Verio; Siemens, Erlangen, Germany) and pelvic phased-array coils. As per the standard mp-MRI in our institution, the images of prostate were obtained on T1-weighted imaging (T1WI), T2WI, a Readout segmentation of long variable echo-trains (RESOLVE) diffusion-weighted imaging (DWI), and follow- ed with dynamic contrast-enhanced (DCE) imaging. | Sensitivity, specificity , LR+, LR- and AUC |
| **Clinical** |  |  |  |  |  |  |  |  |  |  |  |  |  |
| Xiao, 2017 | Retrospective study | internal cross-validation | Private (Hospital) | 941(358,583) | 941(358,583) | Patient-based model | 71[24–88] | N/A | 15.63[ 9.50–47.30] | PCa | N/A | All patients were examined in the left lateral decubitus position using a 5-MHz or 7.5-MHz, convex or linear array biplane rectal probe connected to a PHILIPS HDI color Doppler ultrasound system. | sensitivity, specificity, accuracy, and area under the curve |
| Roffman, 2018 | Retrospective study | internal split-validation | Public | 101705(1672, 100033) | 101705(1672, 100033) | Patient-based model | PCa: 67.01; non PCa: 59.75 | N/A | N/A | PCa | N/A | N/A | Sensitivity, specificity, Accuracy, AUC |
| NOWROOZI, 2016 | Prospective study | internal cross-validation | Private (Hospital) | 699 (221, 448) | 699 (221, 448) | Patient-based model | PCa: 68.4 (8.8) ; non-PCa: 63.9 (7.8) | N/A | PCa: 18.9 (20.5); non-PCa 9.8 (7.2) | PCa | N/A | Serum PSA levels were measured before DRE andTRUS. DRE was performed by experienced urologistsand uro-oncologist and was classiﬁed as normal or sus-picious (any of asymmetry, nodularity, induration orirregularity, etc. ﬁndings was considered as suspicious). TRUS-guided systematic biopsy ofthe prostate was performed using a scanner and 8-MHztransducer. Total TRUS-derived prostate volume (PV) was calculated with the use of prolate ellipse formula(0.52 × length × width × height). All of the biopsieswere performed by an experienced uro-oncologist,and pathologic evaluation was done by experiencedpathologist. Prostate biopsy wasdone by standard systematic 12-core method. Biopsieswere taken with 18-gauge needle biopsy. Furthermore, in patients with suspiciousregions during TRUS evaluation or DRE ﬁndings, additional biopsy cores were taken. | Sensitivity, specificity, AUC |
| Huang, 2018 | Prospective study | internal cross-validation | Private (Hospital) | 231 (75, 156) | 231 (75, 156) | Patient-based model | PCa: 72.87 (6.4); non-PCa: 67.19 (6.60) | N/A | PCa: 22.32 [13.77-36.89]; non-PCa:13.44 [9.44-17.93] | PCa | PZ | MRI was performed using a 1.5T or 3.0T whole-body system (GE Healthcare, USA) and a pelvic array coil (no endorectal coil was used). The imaging protocol included axial T1-weighted images of the pelvis and biplanar T2-weighted fast spin-echo images centred on the prostate. In addition, axial diffusion weighted imaging was performed with b-values of 0, 800, and 1000 sec/mm2. Dynamic contrast-enhanced images were performed following intravenous administration of gadolinium-chelate. | Sensitivity , specificity , and AUC |
| **Genomic** |  |  |  |  |  |  |  |  |  |  |  |  |  |
| Barceló, 2019 | Retrospective study | none | Private (Hospital) | 42 (24, 18) | 42 (24, 18) | Patient-based model | Refer to paper | GS 6: 18; — GS >7: 12 | Refer to paper | PCa and BPH | N/A | Semen samples were obtained by masturbation after 3–5 days of sexual abstinence. They were allowed to liquefy for 30 min at 37 °C. Isolation of exosomal vesicles was per- formed by differential centrifugation steps (1600 xg for 10 min, then 16000 xg for 10 min at 4 °C) including one microfiltration step (0,22 μm pore size) and ultracentrifugation (100000 xg for 2 h at 4 °C) to sediment exosomes. Nanoparticle tracking analysis was performed by NanoSight NS300 (Malvern Instruments Ltd; UK) showing that >90% of the recovered particles had a size of <200 nm. | Sensitivity , specificity , and AUC |
| Roberts, 2015 | Retrospective study | internal cross-validation | Private (Hospital) | 66 (12, 54) | 66 (12, 54) | Patient-based model | PCa: 59 [55–68] ; non-PCa: 64 [59–71] | N/A | PCa : 6.5 [5.13–10.25], non-PCa : 5.8 [3.25–6.95] | PCa and Healthy | N/A | Ejaculate reconstituted in either 20 ml Hanks, subsequently changed to PBS to avoid confounding effects of glucose in parallel metabolomics studies, was layered over 10 ml isotonic Percoll (GE Health- care-Pharmacia) and centrifuged at 974#g for 30– 60min at 4°C and samples were collected in 1ml aliquots, snap-frozen on dry ice and stored at -80°C. The epithelial cell layer at the Percoll inter- face (which appeared as a discrete band in the middle of the test tube with supernatant above and with sperm and non-cellular components at the bottom was then pipetted, washed with 25ml PBS or Hanks and centrifuged at 1258#g for 10min at 4°C. Cells were subsequently subjected to total RNA isolation. | Sensitivity , specificity and AUC |
| Dereziński, 2017 | Retrospective study | internal cross-validation and split-validation | Private (Hospital) | 89 (49, 40) | 89 (49, 40) | Patient-based model | PCa:67.7[52,86]; non-PCa : 61,3 [40,79] | GS 3+3=6 : 19 (38.8); — GS 3+4=7: 20 (40.8);— GS 4+3=7: 4 (8.2 );— GS 4+4=8: 4 (8.2 );— GS 4+5=9: 1 (2.0 );— GS 5+4=9 : 1 (2.0 ). | N/A | PCa and Healthy | N/A | The aTRAQ Kit for Amino Acid Analysis of Physiological Fluids was purchased from Sciex (Framingham, MA, USA). It consisted of amine-modifying labeling aTRAQ reagent Δ8, aTRAQ internal standard set of amino acids labeled with the aTRAQ reagent Δ0, 10 % sulfosalicylic acid, borate buffer of pH 8.5, 1.2 % hydroxylamine and mobile phase modifiers - formic acid and heptafluorobutyric acid. HPLC gradient grade methanol was purchased from J.T. Baker (Center Valley, PA, USA). Deionized water obtained from Millipore Simplicity UV water purification system (Waters Corporation, Milford, MA, USA) was used. | Sensitivity , specificity , accuracy and AUC |
| Santotoribio, 2019 | Prospective study | none | Private (Hospital) | 232 | 232 (32, 200) | Patient-based model | 72 [ 43- 98] | GS ≤7: 30;— GS =8: 2. | Pca: 5.58 [5.19–5.70], non-pca: 6.62 [5.53–7.01] | PCa, healthy | N/A | Prior to biopsy and after obtaining an informed consent, blood specimens were drawn by venipuncture in gel separator serum tubes and centrifuged at 4,000 rpm for 5 minutes. The measurement of the haemolytic index (HI) were determined by colorimetric method on Hitachi Modular cobas c 702 (Roche Diagnostics, Basel, Switzerland) and just the non-haemolysed samples, those with a HI below 50 units, were included in the study. | sensitivity (SN), specificity (SP), Accuracy, AUC |
| Zhang, 2015 | Prospective study | internal split-validation | Private (Hospital) | 580 | 580 (180, 400) | Patient-based model | Training: Pca:68 [55-82], BPH: 68 [55-81], Normal: 68 [55-82]; Testing: Pca:69 [55-83], BPH:69 [55-83], Normal: 69 [55-83] | Training:GS <7: 55 (34.4); — GS=7 (3+4): 42 (26.3); — GS=7 (4+3):33 (20.6) ;— GS  >7: 30 (18.7). Testing: GS <7: 40 (33.3); — GS=7 (3+4): 34 (28.3);— GS=7 (4+3):26 (21.7) ; —GS  >7: 20 (16.7). | Training: PCA: 8.2 [3.2-21.5], BHP:6.3 [2.4-12.3], Normal:3.4 [1.8-6.0]. Testing: PCA: 8.4 [3.4-22.0]; BHP:6.2 [2.5-12.2], Normal: 3.2 [1.6-5.5] | PCa, BPH+ healthy | N/A | The preoperative serum PSA and TR levels were simultaneously measured in the patients using standard methods at admission. Venous blood samples were taken in the morning’s fasting state. After at least 30 min, but within 2 h, the tubes were centrifuged at 20°C for 15 min at 1,200 g, and the sera were stored frozen in plastic vials at -80°C until the time of consecutive analyses. The controls samples were collected and stored in the same way as the PC samples. PSA levels were measured with commercially. Serum levels of TR was measured in duplicate using a solid-phase sandwich ELISA that uses two highly specific antibodies to human TrxR protein (BioVision Incorporated, Milpitas Boulevard, Milpitas, CA, USA) according to the manufacturer’s instruction. available immunoassay methods by DPC Immulite 2000 (Diagnostic Products Corporation, CA, USA). erum levels of TR was mea- sured in duplicate using a solid-phase sand- wich ELISA that uses two highly specific anti- bodies to human TrxR protein (BioVision Incorporated, Milpitas Boulevard, Milpitas, CA, USA) according to the manufacturer’s instruc- tion. The coefficients of variation (CVs) of inter- assay and intra-assay were 4.8-8.0% and 6.2- 9.0%, respectively. | The predictive sensitivity (SEN), specificity (SPE), and , AUC |
| Kirby, 2017 | Retrospective study | external validation | Private (Hospital) and Public Data | 101 | training: 136(73, 63) Testing: 262(213, 49) | lesion-based model | training: 59.9[43–73]; testing: 60.4[43–75] | Training: GS <7: 16; — GS (3 + 4): 40; —  GS (4 + 3): 10; — GS 8: 4; — GS >8: 2; Testing: GS<7: 15; — GS (3 + 4): 84; — GS (4 + 3):50; — GS 8: 25; — GS>8: 39 | training: 6.8 [0.94–42]; testing 10.9[1.6–8]) | PCa | N/A | The QIAGEN AllPrep DNA/RNA mini kit (QIAGEN) was used to extract DNA and RNA. DNA methylation levels by using the Illumina Infinium HumanMethylation 450 K beadchip array (Illumina, San Diego, CA, USA). | sensitivity, specificity, and AUC |
| Patel, 2019 | Retrospective study | external validation | Private (Hospital) | 699 | 795 (699, 96) | lesion-based model | N/A | N/A | N/A | PCa | N/A | DNA was quantified on a Qubit 3.0 Fluorometer (Thermo Fisher Scientific) using the dsDNA HS (High Sensitivity) kit. Individual DNA samples (50 ng) were bisulfite‐converted according to the manufacturer’s protocol (EpiTect Bisulfite Kit; Qiagen). A mastermix was prepared that contained one of 15 primer pairs (400 nM; Thermo Fisher Scientific) and probe sets (150 nM; Thermo Fisher Scientific) (Table S3), nucleotides (250 μM; Invitrogen), MgCl2 (1.2 mM; NEB), stands for Bovine serum albumin (BSA) (0.5 mg/mL; NEB), 6‐carboxy‐X‐rhodamine (ROX) refer- ence dye (24.5 nM; Invitrogen), EpiMark Taq polymerase (0.25 U; NEB) and 1× EpiMark reaction buffer (NEB). Next, bisulfite‐converted DNA (1 μL) was added to the mastermix and 10 μL reactions were amplified using a VIIA7 thermocycler (Applied Biosystems). Cycling conditions included denaturation at 95°C for 30 seconds, seven cycles of touch‐ down PCR with annealing temperatures decreasing by 2°C per cycle, and extension at 68°C for 30seconds, followed by 48 cycles of 30 seconds at 95°C, 30 seconds at 58°C, 30 seconds at 68°C, and a final extension step of 5 minutes at 68°C. | sensitivity (SN), specificity (SP), Accuracy, AUC |
| Amante ,2019 | Retrospective study | internal cross-validation | Private (Hospital) | 91 (43, 48) | 91 (43, 48) | Patient-based model | PCa:70 (10); BPH: 70 ( 8) | GS = 3 + 3: 15; —GS = 3 + 4 and 4 + 3: 21; — GS = 4 + 4 and 4 + 5: 7 | Pca: nd 11.0 (9.5); BPH: 3.8 (2.3). | PCa+BPH | N/A | Tert-butyl methyl ether (TBME), ethyl acetate, dithioerythritol, ammonium iodide (NH4I), N-Methyl-N-(trimethylsilyl)trifluoroacetamide (TMSTFA), and trifluoroacetic anhydride (TFAA) were provided by Sigma-Aldrich (Milan, Italy). β-glucuronidase from Escherichia coli was purchased from Roche Life Science (Indianapolis, IN, USA). Ultra-pure water was obtained using a Milli-Q® UF-Plus apparatus (Millipore, Bedford, MA, USA). | sensitivity, specificity, and area under the curve |
| Brikun, 2019 | Retrospective study | internal cross-validation and split-validation | Private (Hospital) | 94 (42, 52) | 94 (42, 52) | Patient-based model | PCa: 67.1 (7.1); Non-PCa: 63.9 (7.6) | GS 6: 19 (45.2); — GS 7: 8 (19);— GS>7: 13 (30.9) | Pca: 63.9 (7.6); non-PCA: 5.6 (2.7) | Pca+Healthy | N/A | Urine samples were collected using the AssayAssure® urine preservative (Fisher Scientific). DNA was quantitated with the Qubit fluorometer (Life Technologies, Grand Island, NY) using a DNA quantitation kit. | sensitivity, specificity, accuracy, and AUC. |
| Gao, 2019 | Retrospective study | internal cross-validation and split-validation | Private (Hospital). Training cohort from Geisinger Medical Center urologic. Testing cohort from Clinic In- ternal Medicine of El Paso, TX | 183 (108 , 75) | 183 (108 , 75) | Patient-based model | [40-84] | Training: GS 6: 20(36), GS 7: 23(42); — GS 8: 6 (11); — GS 9: 6 (11). Testing: GS 6: 31(58); — GS 7: 16(30); — GS 8: 3 (6); — GS 9: 3(6) | Training: Pca: 5.29 [0.08-1987, non-PCa: 2.6 [0.1-18.2]. Testing: PCA 3.75 [0.08-75], non-Pca 5.21 [1.8-20] | PCa and Healthy | N/A | All chemicals were of analytical grade. Mirex (99.0%, Dr. Ehrenstorfer GmbH, Germany), used as the internal standard, was purchased from the Laboratories of Dr. Ehrenstorfer, Germany. Mirex solution of 100 mg/L was prepared in methanol (liquid chromatography/mass spectrometry grade, Burdick & Jackson, Muskegon, MI). Hydrochloric acid (HCl) (37%) was purchased from Sigma-Aldrich (St. Louis, MO). Ultra-pure deionized (DI) water from Milli-Q system (Millipore, Bedford, MA) was used in the preparation of HCl solution and dilution of urine samples. | sensitivity, specificity, accuracy, and AUC. |
| Donovan, 2015 | Retrospective study | none | Private (Hospital) | 195 | 195 (89, 106) | Patient-based model | All: 62 [42–86] | GS 6: 47 (25); — GS>7: 42 (22) | All: 5.1 [2.1–9.8] | PCa and Healthy | N/A | Using the Urine Clinical Sample Concentrator Kit, exosomes are isolated from the urine with the internal control by ultracentrifugation. The qPCR reactions were performed using the Rotor-Gene Q System (QIAGEN, Venlo, Netherlands). | sensitivity, specificity, PPV, NPV |
| Mengual, 2016 | Retrospective study | internal cross-validation | Private (Hospital) | 224 (151, 73) | 224 (151, 73) | Patient-based model | Pca: 67.5 ( 7.9); Non-PCA: 67.2 ( 12) | GS<7: 69 (46); — GS>7:81(54) | Pca: 13.76 (36.1); non-pca: 1.8  (1.06) | PCa and BPH/Prostatitis | N/A | Voided urine samples (20 to 50 ml including the initial portion of the urine,) were collected following prostatic massage in sterile containers containing 2 ml of 0.5 M EDTA, pH 8.0. Urines were immediately stored at 4 °C and processed within the next 8 h. The samples were centrifuged at 1000xg for 10 min, at 4 °C. The cell pellets were re-suspended in 1 ml of TRIzol reagent (Invitrogen, Carlsbad, CA, USA) and frozen at −80 °C until RNA extraction. RNAs from the urinary cell pellets were extracted using TRIzol reagent (Invitrogen, Carlsbad, CA, USA) according to the manufacturer’s instructions and quantified with a NanoDrop (NanoDrop Technologies, Wilmington, DE, USA). | sensitivity, specificity,NPV, PPV |
| SALIDO-GUADARRAMA, 2016 | Retrospective study | none | Private (Hospital) | 143 (73, 70) | 143 (73, 70) | Patient-based model | Pca: 68 [51–87]; BPH: 65 [50–82] | GS7: 28 (19.6) ; — GS 8: 33 (23.1); — GS 9: 2 (1.4); — GS10: 10 (7.0) | Pca: 15.7 [5–72.4]; BPH: 9.8 [0.9–36] | PCa and BPH | N/A | The collected urine and prostate fluid mixture was mixed with 5 ml RNAlater buffer (Qiagen, Hilden, Germany) as an RNA stabilizing agent. All samples were stored at −80°C for one week. For further processing, samples were defrosted on ice and then centrifuged at 5,000 × g. Once the supernatant was discarded, the urine sediment was washed twice with phosphate-buffered saline at pH 7.2 and RNA extraction was performed using the RNeasy Mini kit (Qiagen, Inc.) according to the manufacturer's guidelines. RNA was re-suspended in 15 µl RNAase-free water and quantified spectrophotometrically using a NanoDrop ND-1000 system (Thermo Fisher Scientific, Inc., Waltham, MA, USA). | sensitivity, specificity, Accuracy, AUC |

**Supplementary Table S4. Characteristics of the performance and validation condition**

| **Author, Year** | **Diagnostic workflow of algorithm** | | | | | | |  |  | **Reference standard of validation dataset** |  |  |  |
| --- | --- | --- | --- | --- | --- | --- | --- | --- | --- | --- | --- | --- | --- |
|  | **Acquisition/Pre-processing** | **Segmentation** | **Extracted (learning) features** | **Method of features selection/extraction** | **Classification algorithm** | **Parameters** | **Predictors** | **VOI/ROI** | **Feature Normalization** | **Validation type** | **Diagnosis criteria of Pca** | **Definition of groups** | **Notes** |
| **Radiomic** |  |  |  |  |  |  |  |  |  |  |  |  |  |
| Chen, 2019 | The study workflow was divided into three steps: manual segmentation, ROI merger, and feature extraction. For consistency between ROIs in both T2WI and ADC images, all depicted ROIs were strictly delineated with the same cri teria and visually validated by the same expert. The whole process of feature extraction was per- formed using Artificial Intelligence Kit software. | Manual segmentation and ROI merging were carried out in consensus by two radiologists using a newly developed software package (Artificial Intelligence Kit, v. 2.0.1, GE Healthcare, China). | Four types of features (first-order statistics, gradient-based histogram features, second-order Haralick textures, and form factor parameters) for a total of 396 features were extracted from ADC images, and 385 features were extracted from T2WI images. | Analysis of variance (ANOVA), Kruskal–Wallis test, univariate logistic, and least absolute shrinkage selection operator (LASSO) were used to explore the informative features . | logistic regression | none | Run High Grey Level Emphasis All Direction, Hara Entropy, Skewness, Long Run Emphasisis All Direction, GLCM Entropy angle, Quantile(0.25) from T2WI and ADC images. | ROI | none | internal-split validation | Biopsy | PCa and Healthy | Authors used SMOTE technique. The Youden index improved from 0.507 to 0.730 on T2WI&ADC with oversampling. |
| Devine, 2019 | Refer to the paper | Manual segmentation by a board-certified radiologist with 5 years of experience in prostate mp-MRI reporting. | ADC and LWF values | none | logistic regression | none | LWI technique, which produces an estimate of the fractional volume of luminal water in each voxel of the prostate, the luminal water fraction (LWF). | ROI | none | internal validation, five‐folds cross validation | Biopsy | PCa patients | N/A |
| Gholizadeh, 2019 | For noise reduction, an adaptive anisotropic diffusion filter was performed using the log-Euclidean anisotropic filter available from the software package, MedINRIA. | Manual segmentation by two radiologists, usingOsiriX software | Quantitative parameters from T2WI, T2WI + DWI, T2WI + DTI (MD + FA) and T2WI + DTI (all DTI quantitative parameters with a significant difference between the two groups). | Criteria of selection: High AUC. | Support Vector Machine (SVM) | Radial basis function (RBF) kernel | Quantitavive parameter of T2W and DTI (all) | ROI | none | leave-one-out cross-validation | Biopsy | PCa patients | Youden indices were used for defining cutoff values of DTI parameters. |
| Le, 2017 | Intensity normalization to rescale intensities of both ADC and T2WI to [0 255]. Additionally, to reduce the intensity differences between the training set and the testing set, the mean intensity of training images was substrected from the intensities of each testing image. To minimize motion-induced misalignment errors, a non-rigid registration method based on mutual information to align ADC data with the corresponding T2WI data was applied. Specifically, the target image (ADC) is gradually deformed according to a free form model (FFD) described by B-splines basis functions until the mutual information between the target image and a reference image (T2WI) is maximized, indicating optimized alignment between the two images. A three-level multi-resolution strategy is applied to search the optimized deformation from coarse to fine to reduce the computational cost and to increase the robustness of the algorithm. | Manual segmentation by two radiologists. | the first-order and second-order features are extracted from manually delineated ROIs from both ADC and T2WI. | A recursive feature selection support vector machine (RFE-SVM) classifier. | multimodal convolutional neural networks (CNNs) | 2D CNN models which are fine-tuned from pre-trained CNN models. ResNet method with 50 layers. | Fusing features from ADC and T2WI | ROI | See acquisition/pre-processing | internal-split validation | Biopsy | PCa patients | N/A |
| Kwon, 2018 | N/A | Manual segmentation by two radiologists. | mp-MRI: 2-W, ADC, BVAL, and Ktrans.216 quantitative imaging variables were ana- lyzed: four modalities (T2-W, ADC, BVAL, and Ktrans) × six features [first-order: intensity (int) and second-order: energy (ene), entropy (ent), correlation (cor), homogeneity (hom), and contrast (con)] × nine descriptors [10%, 25%, 50%, 75%, 90%, mean, standard deviation (SD), kurtosis (kurt), and skewness (skew)]. | Selection was performed by LASSO. | Adaptive least absolute shrinkage and selection operator (LASSO) | none | BVAL_int_SD, T2-W_ene_SD, K trans _ene_Kurt T2-W_ent_90%,  K trans _ene_Skew, K trans _con_Skew, ADC_con_Skew T2-W_ent_Skew T2-W_ene_Skew, BVAL_con_Skew, BVAL_ene_Skew, ADC_ene_Skew, ADC_hom_Skew, BVAL_int_Skew, | ROI | none | 10 fold cross validation | Biopsy | PCa patients | N/A |
| Lay, 2017 | The image filter subtracts the prostate intensity mean from the image and then sets the resulting pixels to 0 when they have an intensity less than or larger than 0, depending on the sequence (T2W, ADC larger than 0 and B2000 less than 0). This follows from the observation that lesions visible in T2W and ADC images have relatively lower intensity than normal prostate tissue and that lesions visible in B2000 tend to have relatively higher intensity. This filter rule thus behaves like a simple cancer classifier in each sequence. | Manual segmentation by two radiologists. | Combination of spatial, intensity, and texture features extracted from three sequences, T2W, ADC, and B2000 images | Randon Forest | Randon Forest | The purity function used in this random forest is the Gini index. This weighted gain is just a generalization of the conventional gain function | N/A | ROI | none | twofold cross-validation | Biopsy | PCa patients | N/A |
| Ma, 2019 | MRI images were submitted to the Siemens syngo® MR B17 workstation. Images were assessed by two senior radiologists and a general consensus was reached for each diagnosis. DCE-MRI quantitative analysis was performed by the same physician using hemodynamics quan- titative analysis Omni-Kinetics software (version 2.1.0.R; GE Healthcare, Shanghai, China) for post processing. | Manual segmentation by one radiologist. | Ktrans, Kep, Ve , and ADC values | Criteria of selection: High AUC. | logistic regression | none | Ktrans + Kep + Ve + ADC | ROI | none | none | Biopsy | PCa and BPH patients | The area under the curve (AUC) was calculated with the cut-off value set at the maximum Youden index: 0.706. |
| Mazaheri, 2019 | Voxel-wise ADC maps were generated on workstations provided by the manufacturer (Advantage Windows; GE Medical Systems) assuming a monoexponential signal decay. | N/A | Voxel-wise ADC maps | none | Cart tree | The Gini index was used to grow the tree, and we set the minimum bucket size to 10 lesions. The optimal tree was displayed in decision tree plot and the decision rule created based on the tree branches. | Voxel-wise ADC maps | ROI | none | 10-fold cross-validation and internal-split testing | Biopsy | PCa patients | N/A |
| Qi, 2019 | N/A | Manual segmentation by two radiologists, using ITK-SNAP software with no prior knowledge of the histopathological results. | A total of 2104 features were extracted from apparent diffusion coefficient (ADC) map, T2WI, and dynamic contrast-enhancing (DCE) MR images according to 1) first-order statistics; 2) shape and size; 3) texture; 4) wavelet filter; and 5) Laplacian of Gaussian (LoG) fil- ter features. Feature extraction was performed based on an open-source Python package (Pyradiomics 2.0.1). | The two-sample Student’s t-test and Pearson correlation coefficient were conducted on the training cohort to select representative features. | multivariate logistic regression | none | the multi-imaging fusion model, age, PSAD, and the PI-RADS v2 score | ROI | none | 10-fold cross-validation and interal-split validation | Biopsy | PCa and Healthy | N/A |
| Reda, 2017 | Refer to the paper | non-negative matrix factorization-based prostate segmentation. | DW-MRI data | N/A | Deep learning: SNCSAE | The whole stacked NCAE classifier (SNCAE) is refined on the input labeled data to obtain better results by backpropagating the error via the entire network and penalizing the softmax layer's negative weights only. The learning parameters a=0.03, b=3, and γ = 0.1, were chosen on the basis of comparative experiments | The CDFs of the refined ADCs at different b-values are considered global water diffusion features and used to distinguish between benign and malignant prostates. | ROI | The ADC map of each subject is described as a whole with a cumulative probability distribution function (CDF) of the normalized ADC for the prostate region. | a leave-one-subject-out (LOSO) cross-validation | Biopsy | PCa patients | N/A |
| Song, 2018 | To reduce variations insignal intensity among different images, all data were normalized before training.Normalization was applied separately to each sequence image of each patient because the contrast difference between images of different sequences is intrinsic and contains useful information for diagnosis. | Manual segmentation by two radiologist. | No feature extraxted, all ROIs were used. The axial T2WI, DWI (b-value of 50, 400, and 800 s/mm2) and ADC maps were used for imaging analysis. | none | Deep Convolutional Neural Network (DNN) | VGGNet with a 1-3-1 layer architecture. The model contained three blocks followed by three fully connected layers. Each block included two convolutional layers with a filter size of 3-3-3, one convolutional layer with a filter size of 1-3-1, and a max-pooling layer with a filter size of 2-3-2. Dropout was performed before max pooling and a fully connected layer to avoid overfitting. Batch normalization was used after each convolutional process. A 1-3 1-layer with two filters followed by soft-max activation at the end of the net was added to obtain the probability map of PCa and non-PCa. ADAM algorithm to update the weights with an initial learn- ing rate of 0.001 and cross-entropy as cost function was used. | T2W-ADC, 2W-ADC-DWI (high b value) | ROI | None | intenal cross validationa and interal-split validation | Biopsy | PCa patients | Youden index was 0.92. |
| Starobinets, 2017 | ADC maps were created using in‐house software, from the combined DWI (b = 600 s/mm2) and T2‐weighted reference images (b = 0 s/mm2) using in house Equation model. | Manual segmentation by two radiologists. | The following combinations of modalities were considered: (i) T2‐weighted imagingand DWI; (ii) T2‐weighted imaging and DCE; (iii) T2‐weighted imaging, DWI, and DCE. | A mixed stepwise logistic regression with a threshold p‐value of 0.15 was used to identify the imaging parameters to be included or excluded in the combined model. | stepwise logistic regression | none | ADC parameter | ROI | none | repeated k‐fold cross‐validation with 10 iterations and k = 4 was performed. | Biopsy | PCa patients | Optimal sensitivity and specificity pairs were chosen from the ROC curve with the assumption that false negatives and false positives come at similar costs. |
| Valerio, 2016 | The data analysis was performed with Olea Sphere software (Olea Medical, LaCiotat, France). | Manual segmentation by two radiologists. | the conventional parameters (T2 and ADC)- the IVIM parameters (D, D* and f)- combined T2-weighted imaging/DWI and IVIM parameters (T2,ADC, D, D* and f). | Criteria of selection: High AUC. | Linear Discriminant Analysis (LDA) | none | T2, ADC, D, D* and f | ROI | none | none | Biopsy | PCa patients | N/A |
| Wang, 2017b | The authors resize the image to 360 × 360 before input the image into deep net- works. | There was no manual work such as lesion-based label annotations or segmentation in the study. | none | none | Deep Convolutional Neural Network (DNN) | A 288 × 288 × 3 MR image was input. Five convolution layers and two inner product layers with sizes were shown in the figure. A max-pooling layer and non-linear ReLU layer following each convolution layer. A max-pooling layer downsize feature map gradually as demonstrated. Finally, an output layer specified PCa patient probability on input image. | axial 2D T2-weighted imaging | none | none | 10-fold cross validation | Biopsy | Pca, BPH  and  prostatitis patients | N/A |
| Wu, 2019 | Patient-identifying information was removed and the axial ADC and T2W-MRI images that depicted the mass where it appeared the larg- est were selected and exported in digital imaging and communica- tions in medicine (DICOM) format from picture archiving and communication system (PACS) to an independent workstation for lesion segmentation. | Segmentation was performed manually using ImageJ. | Quantitative imaging features derived from ADC and T2W- MRI were selected a priori based on the results of previous literature investigating the role of quantitative ADC, T2W-shape and texture analysis. These quantitative features were computed using MaZda v. 4.6 (Institute of Electronics, Technical University of Lodz, Poland).35 | none | logistic regression | none | 10th-centile ADC + T2 W circularity + T2 W entropy + T2 W RLNU + T2 W GLNU | ROI | none | 10-fold cross-validation | Biopsy | PCa and BPH patients | Method of Youden to determine the criterion that corresponded to the maximum accuracy for parametric data. |
| Yang, 2017 | First, the ADC and T2w images are aligned in the spatial domain to correct motion-caused misalignment by a patient’s movement during the image acquisition. Second, an au- tomated method is applied to detect the prostate region using a squared bounding box which encompassing the entire prostate on every T2w slice in the transverse direction. The bounding box is then propagated to all aligned ADC slices. The content within each bounding box is then cropped and the intensities are normalized. | Automated segmentation by CNN. | None | Criteria of selection: High AUC. | co-trained CNN features followed by SVM | The learning rate is set to 10−4 for fine-tuning and 10−3 for training from scratch. For both fine-tuning and training from scratch the learn- ing rate is decayed by 95% for every 1000 iterations. For training from scratch, parameters are initialized using Gaussian distributions. fine-tuning a pre-trained GoogLeNet . | ADC and T2w sequences in mp-MRI data | ROI | none | internal split-validation | Biopsy | PCa and BPH patients | N/A |
| Zhang, 2019 | Package Pyradiomics version 2.0.1 was used to extract radiomic features. | Manual segmentation by two radiologists using itk-SNAP. | 700 radiomic features were extracted automatically based on the delineated ROI. These features could be included into five groups: (I) image intensity features (n = 18), (II) shape and size features (n = 12), (III) texture features (n = 68), and (IV) wavelet-based features (n = 344), and (V) Laplace of Gaussian (LoG) filter-based features (n = 258). | First, features with low variance (less than 0.1) were removed. After that, two sample t test was performed to select features with potential predictive value, and features with p value larger than 0.05 were removed. Next, Redundant features were then removed based on the Pearson correlation coefficient . Finally, least absolute shrinkage and selection operator (LASSO) was used to selected the final predictive features | Random Forest | The parameters of this algorithm were chosen based on the maximal out-of-bag score. | Seven radiomic features with potential predictive value were selected using LASSO with LOOCV. | ROI | z-score method | leave-one-out cross-validation and internal-split validation | Biopsy | PCa and Healthy patients | N/A |
| Zhao, 2015 | Before analysis, T2WI were standardised to correct for background and nonlinearity of the MR image intensity scale. | Manual segmentation by two radiologists. | Quantified features in this study were grouped into three types: general features (mean value, minimum value, standard deviation, coefficient of variation, 10th per- centile), features derived from gray-level histogram (skew- ness, kurtosis), and features derived from co-occurrence matrix (GLCM) (contrast, correlation, energy, homogeneity, entropy) (see Supporting Information). In total, 12 features were extracted from the T2WI. | To select the most informative set of features, sequential forward selection (SFS) was performed with an ANN classifier | Artificial Neural Networks (ANN) | A devised feed forward ANN structure with three layers (input layer, hidden layer, and output layer) was used as a classifier to identify Pca. Back-propagation (BP) algorithm was used in the training, and the Levenberg-Marquardt (LM) algorithm was applied as a learning rule. Different transfer functions were tried in the hidden and output layers, the log-arithmic- sigmoid (Logsig) was finally selected as the transfer func- tion. The network structure with a hidden layer of 14 neu- rons and the epoch number of 500 was used. | six features (sum average, minimum value, SD, 10th percentile, contrast, and correction) re- mained as the inputs of the ANN classifier for PZ. | ROI | none | A leave-one-ROI-out cross-validation approach | Biopsy | PCa and Healthy patients | N/A |
| Bonekamp, 2018 | T2-weighted images and images with b value of 1500 sec/ mm2 were normalized by dividing voxel intensities with the mean value of background PZ tissue. ADC, being a quantita- tive measurement, was not normalized. ADC VOIs were used for analysis of b value of 1500 sec/mm2 images due to the natural coregistration of ADC maps to the source b-value images. Radiomic feature calculations were performed by us- ing the pyradiomics package. | Three-dimensional volumes of interest (VOIs) of clinical lesions were segmented by one investigator. | Within each VOI, (a) 19 first-order features, (b) 16 volume and shape features, and (c) 59 texture features were calculated, leading to 282 fea- tures per VOI. Because these features were calculated on the available ADC maps and on T2-weighted images and images with b value of 1500 sec/mm2, a total of 846 radiomics fea- tures were available for each lesion. Mean ADC was extracted from the radiomic set for separate analysis. | Radiomic features were reduced by univariate feature selection with the remaining features input into random forests (RFs). | Randon Forest | The final RF ensemble comprises 100 forests, with 500 trees each which in turn are grown using a maximum depth of seven, a minimum number of four samples to split a node, using 150 best features according to the feature-selection. | ADC1500_original_firstorder_Maximum, ADC1500_original_firstorder_RootMeanSquared, ADC1500_original_firstorder_Mean, ADC1500_original_firstorder_Median, ADC1500_original_firstorder_10Percentile,DC1500_original_firstorder_90Percentile, ADC1500_original_firstorder_Minimum, T2_original_shape_SurfaceArea, T2_original_shape_MinorAxis, T2_original_shape_Maximun2DDiamiterColumn | VOI | none | interna cross-validation and split validation | Biopsy | PCa patients | N/A |
| Wang, 2017a | Pixel-wised signal intensities (SI) of the prostate on axial T2WI and high-b DWI (1,500 s/mm2) were normalized by dividing them to a mean value of an ROI on the right obturator internus. The DW data were post-processed with a non-Gauss diffusional kurtosis imaging (DKI) model. | The radiologist manually drew volumes of interest (VOIs) of a leading lesion in the peripheral zone (PZ) and/or transitional zone (TZ), with a normal PZ as well as with a TZ focus, respectively, as control, unless no histologically normal region was present | For MR image, eight volumetric MR radiomic features were extracted using a FireVoxel software (Center for Advanced Imaging Innovation and Research [CAI2R], New York University School of Medicine, New York). | In order to optimize the size of a feature set, a pre-diagnostic assessment of feature importance was performed using SVM and recursive feature elimination (SVM-RFE). | Support Vector Machine (SVM) | radial basis function (RBF) kernel | median Dapp (ω = 13.02%), mean Dapp (ω = 12.38%), 90th Kapp (ω = 11.24%) | VOI | Feature values were standardized to a zero mean and unit variance across all samples. | leave-one-out cross-validation approach (LOOCV) | Biopsy | PCa and Healthy patients | A cutoff point was used to maximize the value of the Youden index. |
| **Clinical** |  |  |  |  |  |  |  |  |  |  |  |  |  |
| Xiao, 2017 | none | none | none | Randon Forest | Randon Forest | The final random forest model was built from 500 trees (ntree = 500), and the OOB error rate decreased quickly with increasing tree number until approximately ntree = 50 | age, prostate‐specific antigen levels and transrectal ultrasound findings | none | none | cross-validation (OOB) | Biopsy | PCa and Healthy patients | N/A |
| Roffman, 2018 | None | None | age, body mass index (BMI), diabetes status, smoking status, emphysema, asthma, race (white, black, Native American, Asian, or multiracial),ethnicity (Hispanic or other), hypertension, heart disease,vigorous exercise habits, and history of stroke | none | Artificial Neural Networks (ANN) | This model’s ANN uses two hidden layers with 12 neurons in each layer. A bias variable is intro- duced in the input layer and within each hidden layer. Inputs were normalized to fall in between 0 and 1, and the activation function was always sigmoidal. | age, body mass index (BMI), diabetes status, smoking status, emphysema, asthma, race (white, black, Native American, Asian, or multiracial), ethnicity (Hispanic or other), hypertension, heart disease,vigorous exercise habits, and history of stroke | none | Inputs were normalized to fall in between 0 and 1. | internal split-validation | Biopsy | PCa and Healthy patients | N/A |
| NOWROOZI, 2016 | none | none | age, prostate‐specific antigen (PSA), free/total PSA (%fPSA), digital rectal examination (DRE) findings, prostate volume (PV) and presence of hypoechoic lesion on TRUS | Criteria of selection: High AUC. | logistic regression | none | age, prostate‐specific antigen (PSA), free/total PSA (%fPSA), digital rectal examination (DRE) findings, prostate volume (PV) and presence of hypoechoic lesion on TRUS | none | none | internal validation, using bootstrap technique | Biopsy | PCa and Healthy patients | N/A |
| Huang, 2018 | none | none | Monogram: Age, PSA, free/total PSA ratio; PV, prostate volume; PSAD, PSA density; PSAV, PSA velocity; DRE, digital rectal examination; TRUS, transrectal ultrasound; mpMRI, multi-parametric magnetic resonance imaging. | none | logistic regression | none | Monogram: Age, PSA, free/total PSA ratio; PV, prostate volume; PSAD, PSA density; PSAV, PSA velocity; DRE, digital rectal examination; TRUS, transrectal ultrasound; mpMRI, multi-parametric magnetic resonance imaging. | none | none | internal validation, using bootstrap technique | Biopsy | PCa and Healthy patients | N/A |
| **Genomic** |  |  |  |  |  |  |  |  |  |  |  |  |  |
| Barceló,2019 | First-stranded cDNA specific for miRNA was obtained by RT of 50 ng of exosomal RNA in 10 μl, using the Universal cDNA synthesis kit II (Exiqon; Denmark). For qPCR analysis, cDNA was diluted (12 × ) and assayed in 10 μl PCR reactions containing ExiLENT SYBR Green master mix (Exiqon; Denmark). Duplicate amplification reactions of individual assays (LNATM-enhanced miRNA qPCR primers) were carried out on a Lightcycler® 96 Instrument (Roche; Switzerland). Target miRNA expression for exosomes in semen samples was calculated relative to the mean expression value of miR-30e-3p and miR-126-3p, chosen from the exosomal miRNA qPCR profiling study as being among the most stable miRNAs . The RQ values were calculated using the 2dCp strategy. The same procedure was applied to determine tissue expression profiling of miRNA candidates with some modifications: 20 ng of tissue RNA were reverse transcribed and cDNA was diluted 80 × . | N/A | miRNA levels | multivariate binary logistic regression analysis was used for selection of the optimal combination of variables. | binary logistic regression | backward stepwise | PSA, miR142-3p, miR-142-5p | N/A | To correct for potential overall differences in amount and quality between the samples, the raw data (Crossing Points Cp values) were normalised for each sample to the mean of the 50 most stable assays (mean 50) that were detected in all samples: dCp = mean 50 Cp – assay Cp. | none | biopsy | Pca and BPH patients | The threshold value was determined by Youden’s index, calculated as sensitivity plus specificity-1. |
| Roberts, 2015 | Ejaculate reconstituted in either 20 ml Hanks subsequently changed to PBS to avoid confounding effects of glucose in parallel metabolomics studies, was layered over 10 ml isotonic Percoll (GE Health- care-Pharmacia) and centrifuged at 974#g for 30– 60min at 4°C and samples were collected in 1ml aliquots, snap-frozen on dry ice and stored at -80°C. The epithelial cell layer at the Percoll inter- face (which appeared as a discrete band in the middle of the test tube with supernatant above and with sperm and non-cellular components at the bottom was then pipetted, washed with 25ml PBS or Hanks and centrifuged at 1258#g for 10min at 4°C. Cells were subsequently subjected to total RNA isolation. Hepsin and PCA3 scores were calculated with reference to PSA in ejaculate. A Hepsin: PCA3 ratio was also calculated, following preliminary analysis that indicated the two markers (relative to PSA-PCR) to be inversely associ- ated (see Results). The qPCR results were analysed using the three clinical classifications. | N/A | serum PSA, PCA3, Hepsin, Hepsin/PCA3, and miR’s (miR-200c, miR-200b, miR-375, and miR-125b) from ejuculate samples. | Stepwise regression | Stepwise regression | none | PSA and Hepsin/PCA3 | none | N/A | BCa bootstrap interval with 1000 iterations | Biopsy | PCa and Healthy patients | Specificity was evaluated by BCa bootstrap interval with 1000 iterations at 90% Sensitivity. |
| Dereziński, 2017 | The analysis of free amino acid profiles in serum and urine was based on the LC-ESI-MS/MS technique and the aTRAQ reagent. The aTRAQ kit allows to quantify 42 free amino acids, both proteinogenic and non-proteinogenic, in a range of biological fluids. The analyses were performed using the liquid chromatography instrument 1260 Infinity (Agilent Technologies, Santa Clara, CA, USA) coupled to the 4000 QTRAP mass spectrometer (Sciex, Framingham, MA, USA) with an electrospray ion source. | none | 42 proteinogenic and non-proteinogenic amino acids in two different physiological fluids obtained from the same groups of prostate cancer patients and healthy men.The variables in the analyses were the amino acid concentrations quantified in serum and urine samples. | variable importance in projection (VIP) scores | PLS-DA | none | methionine, 3-methylhistidine, serine, sarcosine and proline from serum | none | none | Monte-Carlo cross validation and internal-split validation | Biopsy | PCa and Healthy patients | N/A |
| Santotoribio, 2019 | Serum biomarkers were measured: PSA and free-PSA by electrochemiluminescence immunoassay on Hitachi Modular E-170 analyzer (Roche Diagnostics, Basel, Switzerland); LDH by enzymatic photometric method according to the International Federation of Clinical Chemistry and CRP by immunoturbidimetric test with monoclonal anti-CRP antibodies on Hitachi Modular cobas c 702 analyzer (Roche Diagnostics, Basel, Switzerland). | N/A | serum PSA and CRP and Serum LDH levels and %fPSA | Relevance analysis by analysis of variance test for normally distributed variables and Mann- Whitney test for variables with non-Gaussian distribution. | Logistic regression | none | Serum LDH levels and %fPSA | none | none | none | Biopsy | PCa and Healthy patients | The optimal cut-off point was that which had the highest sensitivity and specificity, which correctly classified the largest number of patients. |
| Zhang, 2015 | PSA levels were measured with com- mercially available immunoassay methods by DPC Immulite 2000 (Diagnostic Products Corporation, CA, USA). A cut-off value of 4 ng/ mL was used. Serum levels of Thioredoxin reductase (TR) was mea- sured in duplicate using a solid-phase sand- wich ELISA that uses two highly specific anti- bodies to human TrxR protein (BioVision Incorporated, Milpitas Boulevard, Milpitas, CA, USA) according to the manufacturer’s instruction. | none | PSA and TR | none | logistic regression | none | PSA and TR | none | none | external validation | Biopsy | PCa, BPH and healthy patients | N/A |
| Kirby, 2017 | The authors assayed DNA methylation levels by using the Illu- mina Infinium HumanMethylation 450 K beadchip array (Illumina, San Diego, CA, USA) and calculated the methylation beta score as: b = IntensityMethylated/(Inten- sityMethylated + IntensityUnmethylated). We converted data points that were not significant above background inten- sity to NAs. We removed CpGs having greater than 10% missing values prior to normalization. Data was normal- ized with the ComBat R package. Post-ComBat normalization, we observed that the Infinium I and II as- says showed two distinct bimodal b-value distributions, so we developed a regression method to convert the type I and type II assays to a single bimodal b-distribution corresponding to Reduced Representation Bisulfite Se- quencing (RRBS) b-values . After the Methylation 450 K data was converted to RRBS b-values, any values less than zero were assigned zeros and values greater than one were assigned ones. | None | genome-wide DNA methylation patterns | criteria of selection: High AUC. | logistic regression | none | 3 CpGs:cg00054525, cg16794576, and cg24581650 | none | Post-ComBat normalization | external validation | Biopsy | PCa Patients | N/A |
| Patel, 2019 | The relative threshold method, Crt (Applied Biosystems Relative Quantification “RQ” application on Thermo Fisher Scientific Cloud) was used to determine cycle quantification (Cq) values for each amplification curve. Crt parameter optimization (early access version, Thermo Fisher Scientific Cloud) was conducted to enhance the reliable detection of amplification. Sample reactions with inconclu- sive amplification curves, contamination, or poor reaction efficiency were excluded from further analysis. Reactions with negative amplification were assigned a Cq two higher than the maximum observed Cq value in their respective cohort. | none | all possible combinations of all 15 DNA methylation regions | criteria of selection: High AUC. | logistic regression | none | GAS6/GSTP1/HAPLN3 | none | Normalized methylation levels were calculated using ΔΔCt method. | external validation | Biopsy | PCa Patients | N/A |
| Amante, 2019 | The PARADISe software, coupled with NIST libraries, was employed for the computation of PARAFAC2 models, the extraction of the significative components (alleged biomarkers), and the generation of a semiquantitative dataset. | N/A | The preliminary PARAFAC2 model extracted a total of 329 relevant compounds (184 from the  chromatographic run after TMS derivatization and 145 after TFA derivatization). | Variable importance in projection (VIP) method (using a threshold of 1) and genetic algorithms (GAs). | PLS-DA | N/A | 32 urinary biomarkers | N/A | The dataset was log10 transformed and autoscaled. | repeated double cross-validation (dCV) approach | Biopsy | Pca and BPH patients | N/A |
| Brikun, 2019 | The assays were designed from portions of the CpG islands that allowed for the selection of two primary amplification primers, a Taqman hydrolysis probe, and at least two amplification primers. The primary amplification primers were separated by < 200 bp and preferably contained no CpGs or at most a single CpG dinucleotide. The Taqman hydrolysis probe contained at least three CpGs and the PCR amplification primers preferably contained two or more CpGs. The conditions of the bisulfite conversion and subsequent amplifications were optimized for 10 ng of input DNA. The length of the bisulfite treatment was determined blindly using a training set of 10 urine DNAs selected from the urine samples collected for this study. Three or more bisulfite time points were performed on 10 ng of the training set DNAs to select the best conditions for the deamination of individual markers as well as groups of markers. Markers were grouped into two bisulfite conditions, (14minat70°Cand42minat80°C)basedonthere- sults obtained with the training set. Ten nanograms of urine DNA (a mix of sediment and supernatant DNA) were used for each bisulfite reaction. | N/A | Methylation markers from urine and clinical variables such as age and PSA | Methylation markers and clinical variables such as age and PSA were subject to variable selection by the algorithms. | elastic net and logitboost | Tuning Method: Max_AUC | The panel of markers is composed of 19 CpG islands associated with 18 genes. | N/A | none | fivefold cross-validation approach and internal-split validation | Biopsy | PCa and Healthy patients | The top-performing models were ranked based on the area under the ROC curve (AUC) or Youden’s Index in the test sets. |
| Gao, 2019 | Urinary VOCs were analyzed in a thermal desorption unit (TDU, Gerstel), coupled with a 6890-GC system and a 5973-N Mass Selective Detector (Agilent Technologies, Wilmington DE). The thermal desorption process was programmed as follows. The initial temperature was set at 45 C holding for 0.5 minutes; the temperature was increased to 300 C at 60 C/minute and held for 5 minutes. Desorption gas flow was set at 1.0 mL/minute. During desorption, all the desorbed compounds were concentrated in a cold injection system, CIS4 (Gerstel), at "40C prior to GC injection. Once the desorption process was completed, the CIS4 was heated to 300 C at 12 C/second and held for 5 minutes in a solvent vent mode. The VOCs were separated and analyzed by GC-MS using splitless mode through a ZB-5ms capillary column (30 m % 0.25 mm % 0.25 mm; Phenomenex, Torrence, CA). The authors used Mirex as the internal standard because of its nonexis- tence in human urine. The relative intensity of each VOC peak could then be normalized against that of Mirex, allowing semi- quantitative statistical analysis of VOCs. | N/A | A total of 9144 potential VOCs were detected | LASSO | regularized logistic regression models with LASSO | none | 11 VOCs | none | none | 10-fold cross-validation | Biopsy | PCa and Healthy patients | The optimal cut point for the diagnostic model was calculated by the maximum Youden Index. |
| Donovan, 2015 | The qPCR reactions were performed using the Rotor-Gene Q System (QIAGEN, Venlo, Netherlands). For each reaction, a Ct (cycle threshold) value was derived for each biomarker and the internal control (Rotor-Gene Q User Manual, November 2012). Calibrators for each target gene plus the internal control were in each qPCR run and used to convert Ct-values to RNA copies. Calibration curves were generated for each qPCR plate to adjust for inter-run variability. Samples were normalized for RNA levels with SPDEF (SAM-pointed domain-containing Ets transcription factor) to derive ‘ERG or PCA3 RNA copy number/SPDEF mRNA copy number.’ The performance of the EXO score using PSA RNA (encoded by kallikrein-related peptidase 3 (KLK3)) was also evaluated using receiver operating characteristics analysis. The area under the curve (AUC) resulting from the use of KLK3 as a normalizer was comparable, with that of SPDEF being slightly improved. | N/A | EXO106 from Urine sample and (SOC) parameters (that is, serum PSA levels, age, race and family history of PCa ) | Criteria of selection: High AUC. | logistic regression | none | EXO106 and (SOC) parameters (that is, serum PSA levels, age, race and family history of PCa ) | None | None | none | Biopsy | PCa and Healthy patients | Specificity was evaluated by BCa bootstrap interval with 1000 iterations at 90% Sensitivity. |
| Mengual, 2016 | The real-time qPCR analysis software was used to obtain cycle quantification (Cq) values. Threshold was manually calculated for each gene. Since experimental errors such as inaccurate pipetting or contamination can result in amplification curves that look significantly different from a typical amplification curve, all amplification plots were checked both computationally and manually. Relative expression levels of target genes within a sample was expressed as ΔCq (ΔCq = Cqendogenous control-Cqtarget gene). We used as endogenous control the mean Cq value of KLK2 and KLK3, which allowed us to normalize the pros- tate epithelial cell content in the collected urine sample. | N/A | 42 genes | LASSO | logistic regression | none | HIST1H2BG, SPP1, ELF3 and PCA3 | none | none | leave-one-out cross-validation (LOOCV) and 100 randomisations with 5- fold cross-validation (5fCV) | Biopsy | PCa, BPH and Prostatitis patients | N/A |
| SALIDO-GUADARRAMA, 2016 | For the validation study and time course analysis, RT, pre‐amplification and qPCR reactions were performed. In order to avoid group bias, the cycle threshold (Ct) distributions for all PCa and BPH samples were determined and a filtering procedure was applied to classify the miRNAs according to their level of detectability. A Ct value of 35 was set arbitrarily; miRNAs with Ct<35 were considered as detected and those with Ct>35 were considered undetected. miRNAs that were undetected in >2 samples for each group were removed from the analysis. Filtered raw Ct data were normalized using a rank-invariant set normalization procedure. Briefly, this normalization computes all rank-invariant sets of features between pair-wise comparisons of each samples against a pseudo-mean | N/A | The expression of let-7c, miR-193b, miR-100, miR-200b, miR-200c, miR-20a, miR-204, miR-195, miR-150, miR-16, miR-21 and let‐7b was measured using TaqMan® MicroRNA Assays (cat. no. 4427975; Thermo Fisher Scientific, Inc.). For measurement of GAPDH and KLK3 mRNA expression levels, Single Tube TaqMan® Gene Expression Assays | Univariable logistic regression | Multivariable logistic regression | none | Age, PSA, %free PSA, DRE, mir-100/200b | none | See acquisition/pre-processing | none | Biopsy | PCa and BPH patients | N/A |

**Supplementary Table S5. PRISMA checklist.**

| **Section/topic** | **#** | **Checklist item** | **Reported on page #** |
| --- | --- | --- | --- |
| **TITLE** | | |  |
| Title | 1 | Identify the report as a systematic review, meta-analysis, or both. | Page 1 |
| **ABSTRACT** | | |  |
| Structured summary | 2 | Provide a structured summary including, as applicable: background; objectives; data sources; study eligibility criteria, participants, and interventions; study appraisal and synthesis methods; results; limitations; conclusions and implications of key findings; systematic review registration number. | Page 1 |
| **INTRODUCTION** | | |  |
| Rationale | 3 | Describe the rationale for the review in the context of what is already known. | Page 2 and 3 |
| Objectives | 4 | Provide an explicit statement of questions being addressed with reference to participants, interventions, comparisons, outcomes, and study design (PICOS). | Page 3 |
| **METHODS** | | |  |
| Protocol and registration | 5 | Indicate if a review protocol exists, if and where it can be accessed (e.g., Web address), and, if available, provide registration information including registration number. | Not Available |
| Eligibility criteria | 6 | Specify study characteristics (e.g., PICOS, length of follow-up) and report characteristics (e.g., years considered, language, publication status) used as criteria for eligibility, giving rationale. | Page 3, and supplementary materials |
| Information sources | 7 | Describe all information sources (e.g., databases with dates of coverage, contact with study authors to identify additional studies) in the search and date last searched. | Page 3, and supplementary materials |
| Search | 8 | Present full electronic search strategy for at least one database, including any limits used, such that it could be repeated. | Page 3, and supplementary materials |
| Study selection | 9 | State the process for selecting studies (i.e., screening, eligibility, included in systematic review, and, if applicable, included in the meta-analysis). | Page 3, 4, and supplementary materials |
| Data collection process | 10 | Describe method of data extraction from reports (e.g., piloted forms, independently, in duplicate) and any processes for obtaining and confirming data from investigators. | Page 4 and supplementary materials |
| Data items | 11 | List and define all variables for which data were sought (e.g., PICOS, funding sources) and any assumptions and simplifications made. | Page 4, 5 and supplementary materials |
| Risk of bias in individual studies | 12 | Describe methods used for assessing risk of bias of individual studies (including specification of whether this was done at the study or outcome level), and how this information is to be used in any data synthesis. | Page 4, and Supplementary material |
| Summary measures | 13 | State the principal summary measures (e.g., risk ratio, difference in means). | Page 4 and 5 |
| Synthesis of results | 14 | Describe the methods of handling data and combining results of studies, if done, including measures of consistency (e.g., I^2^) for each meta-analysis. | Page 4 and 5 |

| **Section/topic** | **#** | **Checklist item** | **Reported on page #** |
| --- | --- | --- | --- |
| Risk of bias across studies | 15 | Specify any assessment of risk of bias that may affect the cumulative evidence (e.g., publication bias, selective reporting within studies). | Supplementary material |
| Additional analyses | 16 | Describe methods of additional analyses (e.g., sensitivity or subgroup analyses, meta-regression), if done, indicating which were pre-specified. | Page 4 and 5 |
| **RESULTS** | | |  |
| Study selection | 17 | Give numbers of studies screened, assessed for eligibility, and included in the review, with reasons for exclusions at each stage, ideally with a flow diagram. | Page 5 and 6 |
| Study characteristics | 18 | For each study, present characteristics for which data were extracted (e.g., study size, PICOS, follow-up period) and provide the citations. | Page 5, 6 and supplementary material |
| Risk of bias within studies | 19 | Present data on risk of bias of each study and, if available, any outcome level assessment (see item 12). | supplementary material |
| Results of individual studies | 20 | For all outcomes considered (benefits or harms), present, for each study: (a) simple summary data for each intervention group (b) effect estimates and confidence intervals, ideally with a forest plot. | Page 5 to 14 |
| Synthesis of results | 21 | Present results of each meta-analysis done, including confidence intervals and measures of consistency. | From page 5 to 14 |
| Risk of bias across studies | 22 | Present results of any assessment of risk of bias across studies (see Item 15). | supplementary material |
| Additional analysis | 23 | Give results of additional analyses, if done (e.g., sensitivity or subgroup analyses, meta-regression [see Item 16]). | From page 5 to 14 |
| **DISCUSSION** | | |  |
| Summary of evidence | 24 | Summarize the main findings including the strength of evidence for each main outcome; consider their relevance to key groups (e.g., healthcare providers, users, and policy makers). | Page 14, 15, 16 |
| Limitations | 25 | Discuss limitations at study and outcome level (e.g., risk of bias), and at review-level (e.g., incomplete retrieval of identified research, reporting bias). | Page 17 |
| Conclusions | 26 | Provide a general interpretation of the results in the context of other evidence, and implications for future research. | Page 16, 17 |
| **FUNDING** | | |  |
| Funding | 27 | Describe sources of funding for the systematic review and other support (e.g., supply of data); role of funders for the systematic review. | Page 18 |

*From:*  Moher D, Liberati A, Tetzlaff J, Altman DG, The PRISMA Group (2009). Preferred Reporting Items for Systematic Reviews and Meta-Analyses: The PRISMA Statement. PLoS Med 6(6): e1000097. doi:10.1371/journal.pmed1000097
